# Impact of the Experimental Parameters on Catalytic Activity When Preparing Polymer Protected Bimetallic Nanoparticle Catalysts on Activated Carbon

**DOI:** 10.1021/acscatal.1c05904

**Published:** 2022-03-30

**Authors:** Charlie B. Paris, Alexander G. Howe, Richard James Lewis, Daniel Hewes, David J. Morgan, Qian He, Jennifer K. Edwards

**Affiliations:** † Cardiff Catalysis Institute (CCI), School of Chemistry, Cardiff University, Main Building, Park Place, Cardiff CF10 3AT, U.K.; ‡ Department of Materials Science and Engineering, Faculty of Engineering, National University of Singapore, Blk E2, #05-01, 9 Engineering Drive 1, 119077 Singapore; § Max Planck Centre for Fundamental Heterogeneous Catalysis (FUNCAT), Cardiff Catalysis Institute, School of Chemistry, Cardiff University, Main Building, Park Place, Cardiff CF10 3AT, U.K.; ∥ HarwellXPS-the EPSRC National Facility for Photoelectron Spectroscopy, Research Complex at Harwell (RCaH), Didcot, Oxon. OX11 0FA, U.K.

**Keywords:** carbon characterization, sol immobilization, nanoparticles, gold−palladium catalyst, hydrogen peroxide direct synthesis

## Abstract

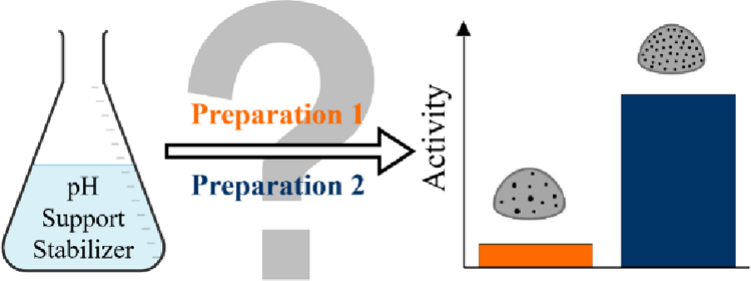

Sol immobilization is used to produce bimetallic catalysts with higher activity to monometallic counterparts for a wide range of environmental and commercial catalytic transformations. Analysis of complementary surface characterization (XPS, Boehm’s titration, and zeta potential measurements) was used to elucidate alterations in the surface functionality of two activated carbon supports during acid exposure. When considered in parallel to the experimentally determined electrostatic and conformational changes of the polymer surrounding the nanoparticles, an electrostatic model is proposed describing polymer protected nanoparticle deposition with several polymer–carbon support examples described. Consideration of the electrostatic interactions ensures full deposition of the polymer protected nanoparticles and at the same time influences the structure of the bimetallic nanoparticle immobilized on the support. The normalized activity of AuPd catalysts prepared with 133 ppm H_2_SO_4_ has a much higher activity for the direct synthesis of hydrogen peroxide compared to catalysts prepared in the absence of acid. Detailed characterization by XPS indicates that the surface becomes enriched in Au in the Au–Pd samples prepared with acid, suggesting an improved dispersion of smaller bimetallic nanoparticles, rich in Au, that are known to be highly active for the direct synthesis reaction. Subsequent microscopy measurements confirmed this hypothesis, with the acid addition catalysts having a mean particle size ∼2 nm smaller than the zero acid counterparts. The addition of acid did not result in a morphology change, and random alloyed bimetallic AuPd nanoparticles were observed in catalysts prepared by sol immobilization in the presence and absence of acid. This work shows that the deposition of polymer protected AuPd nanoparticles onto activated carbon is heavily influenced by the acid addition step in the sol immobilization process. The physicochemical properties of both the polymer and the activated carbon support should be considered when designing a bimetallic nanoparticle catalyst by sol immobilization to ensure the optimum performance of the final catalyst.

## Introduction

Bimetallic nanoparticle catalysts show a synergy in activity when compared to their monoatomic counterparts and are used ubiquitously in heterogeneous catalysis, with thousands of articles published in the last 2 decades. The interaction between the two metals in a bimetallic nanoparticle often confers improved performances (activity, selectivity, and/or stability) in the bimetallic nanoparticles compared to their monometallic counterparts.^1,2^ Bimetallic catalysts are utilized in applications in various industrially relevant fields such as energy (i.e., MoW or CoNi for the hydrodesulfurization of hydrocarbon fuels),^3^ transport (i.e., PtRh in catalytic converters),^4^ or environment (i.e., for AuPd for water disinfection).^5^ Nanoparticle catalysts are often prepared via precipitation of metal precursors onto catalyst supports through the addition of a base,^6^ direct impregnation of precursors onto a support followed by a thermal/chemical treatment,^7^ or preformed synthesis of a polymer protected sol that can be subsequently deposited on a support. The latter process, sol immobilization, allows for the manipulation of the structure of the nanoparticle (alloy/core–shell and size) before deposition, in principle resulting in a well-dispersed bimetallic catalyst with well-defined physical characteristics. However, there can be discrepancies in the structure of the preformed sol and the immobilized nanoparticles, and the fundamental chemistry involved in the deposition process is often poorly reported with experimental parameters followed from prior literature protocols verbatim, with little consideration of the electrostatic or chemical changes that underpin and control the deposition process and the resulting nanoparticle structure. Herein we describe how the chemistry of the support and polymer is heavily influenced by the acid addition step in the sol immobilization process that influences not only the fraction of bimetallic nanoparticles deposited but also the structure of the nanoparticle on the support and the resulting catalytic activity. In this case, we have chosen Au–Pd bimetallic nanoparticles and the direct synthesis of H_2_O_2_ from H_2_ and O_2_ as the exemplar catalytic reaction.

Recently, significant attention has been given to AuPd-supported catalysts for the direct synthesis of hydrogen peroxide from molecular oxygen and hydrogen.^8^ This reaction appears as an eco-friendly alternative to the highly energy-intensive anthraquinone process, which largely dominates the global production of H_2_O_2_.^9^ Pd-based catalysts have been widely studied for the reaction because of their high activity.^10^ However, these monometallic catalysts suffer from poor selectivity as they also facilitate both the hydrogenation and decomposition of the product.^11^ Alloying Au with Pd greatly inhibits this selectivity problem. Bimetallic AuPd catalysts are more active and more selective than their monometallic counterparts.^12−14^ The origin of the synergy of Au–Pd alloys is still under debate but is likely a combination of electronic, structural, and isolation effects.^8^ The synergy between Au and Pd has been verified on a large variety of supports, with carbon-based materials emerging as good candidates due to their intrinsic acidity.^15^ AuPd/C catalysts have been reported to be more active than their homologues supported on metal oxide supports, such as TiO_2_ or Al_2_O_3_.^14,15^

Several parameters play an important role in determining the activity of supported AuPd nanoparticles. Randomly alloyed nanoparticles with a small and narrow particle size distribution are desirable to maximize the overall yield of hydrogen peroxide.^16^ Unlike the sol immobilization technique, other conventional preparation procedures do not allow for good control over nanoparticle characteristics.^17^

The sol immobilization preparation technique is based on the immobilization of a preformed metal sol on a support, allowing—in theory—for the capability to tune the size and shape of the nanoparticles on any chosen support.^18^ During the preparation, dissolved metals are mixed with a stabilizer (polymer, surfactant) and then reduced to form suspended metallic nanoparticles. Subsequently, the addition of the support yields the immobilized heterogeneous catalyst. The manipulation of reaction parameters, such as the nature and the concentration of both the reducing agent and stabilizer,^19,20^ the addition order of the reagents,^21,22^ or any subsequent heat treatment,^16,23^ allows some control over the resulting particle size and elemental distribution (for bimetallics), and these can have significant effects on the physicochemical properties of the final material and hence its catalytic performances.^20^

Dimitratos *et al.* showed the importance of the nature of the reductant; when NaBH_4_ was used, small AuPd nanoparticles (>2 nm) were observed, whereas when N_2_H_4_ was used, larger AuPd nanoparticles (6.1 nm) were obtained.^24^ The nature of the reducing agent influences the size of the nanoparticles: typically, the stronger the reductant, the smaller the nanoparticles.^25^ As most catalytic reactions are size-dependent,^26^ for a given catalytic application, an appropriate reducing agent capable of producing supported nanoparticles of the required size should be used. This follows through the choice of surfactant, volume of acid added, support used, and temperature of the reaction. However, many studies select preparation conditions arbitrarily, overlooking the chemistry taking place during the preparation.

Here, we show that parameters for the preparation of supported metal nanoparticles *via* a sol immobilization method should be carefully considered before an experimental regimen is followed. Specifically, the addition of acid should be tailored to the support–stabilizer combination and the final application of the catalyst. We found that adding acid to the sol modifies the electrostatic interactions between PAA-stabilized AuPd nanoparticles and carbon-based supports, leading to an increased metal immobilization fraction. Moreover, the acid addition favors the formation of small Au-rich nanoparticles on said supports, increasing the final metal dispersion. In combination, these two effects lead to a significantly increased catalytic activity. We demonstrate this approach for PAA-stabilized bimetallic AuPd nanoparticles supported on two different carbons and extend it to a series of stabilizers. The influence of the preparation parameters on the performances of our catalysts has been evaluated toward the direct synthesis of hydrogen peroxide from molecular hydrogen and oxygen and its subsequent degradation. We foresee this approach to be relevant for the preparation of enhanced catalysts designed for many applications requiring supported bimetallic nanoparticles.

## Experimental Section

### Catalyst Preparation

The sol immobilization protocol is based on a methodology previously reported in the literature.^27,28^ The procedure below outlines the methodology for producing 2 g of 0.5 wt % Au–0.5 wt % Pd/C: Aqueous solutions of PdCl_2_ (6 g L^–1^, Sigma Aldrich) and HAuCl_4_·3H_2_O (12.25 g L^–1^, Strem Chemicals) were prepared. Requisite amounts of both Au and Pd solutions (0.816 and 1.666 mL, respectively) were added to deionized water (800 mL) under vigorous stirring at room temperature. For other Au/Pd ratios, appropriate amounts of the precursor solutions were used. The stabilizer (PAA, PVA, SPSS, or PDDA) was added as a 1 wt % aqueous solution to reach a monomer/metal molar ratio of 1.15. The resulting solution was stirred for 2 min before the addition of freshly prepared NaBH_4_ (0.1 mol L^–1^, Acros Organic) aqueous solution such that the molar ratio of NaBH_4_/metals was equal to 5. The solution was then vigorously stirred for 0.5 h before the addition of the support (1.99 g). The solution was acidified to pH 2 with H_2_SO_4_ (98 wt %, Fisher Scientific) (if applicable) and stirred for 1 h. The suspension was then filtered under a vacuum and washed with deionized water until a neutral pH was reached. The resulting catalyst was dried for 16 h at 110 °C before use. An analogous preparation method was used for all catalysts. Two different supports were used: graphene nanoplatelets (Alfa Aesar) and carbon black (KBB, Norit). Catalysts are named as follows: 1%AuPd/Support if no acid was added and 1%AuPd/Support-H^+^ if acid was added during the preparation.

A similar procedure was followed for the preparation of the colloidal suspension, apart from the support addition.

### Characterization

#### Carbon Titration

Quantification of oxygenated surface groups was based on a procedure described elsewhere.^29^ Carbons were pretreated as follows: 1 g of carbon was dispersed in 200 mL of deionized water using an ultrasound bath for 0.5 h. The suspension was then flushed with N_2_ for 1 h to remove any traces of dissolved CO_2_. The sample was centrifuged (10 min, 4350 rpm), and the supernatant was tested with a pH indicator. This procedure was repeated until neutral pH was reached. Then, the sample was dried at 130 °C in static air for 24 h. The dry sample was crushed and sieved (250 μm) to provide small particles that are easily dispersible. Sample titration was performed as follows: *ca.* 0.3 g of pretreated samples was suspended in freshly prepared 0.01 M NaOH (Scientific Laboratory Supplies), Na_2_CO_3_ (Fisher Scientific), or NaHCO_3_ (Fisher Scientific) solutions and stirred for 72 h. The suspension was filtered using a PTFE syringe filter (450 nm). Ten milliliters of the filtrate was mixed with 20 mL of HCl (0.01 M) and back titrated with 0.005 M Na_2_CO_3_ on a pH autotitrator (Metrohm). Before use, Na_2_CO_3_ was dried for 24 h at 130 °C to remove any traces of hydrates. Three batches of each base-carbon suspension were analyzed. Titrations of the filtrates were performed in triplicate. Titration of the references and the HCl solutions was performed five times. Reported results correspond to the average obtained for each batch.

#### DLS

Dynamic light scattering (DLS) analysis was performed on a Zetasizer Nano ZS (Malvern Instruments Ltd., England) equipped with a 633 nm ″red″ laser. Backscattered light was detected at 173°. Measurements were achieved on diluted (1:10 v/v) colloidal suspensions to ensure both a correlation function intercept and a count range in an acceptable range. All samples were run at least three times.

#### Gas Physisorption

N_2_-physisorption analyses were performed on a Micromeritics 3Flex instrument at 77 K. Prior to analysis, *ca.* 0.15 g of the sample was degassed at 200 °C under a vacuum for 24 h. Free-space was measured post-analysis using He. The Brunauer–Emmett–Teller (BET) model was used to determine the specific surface area under the relative pressure range of 0.05–0.30. Total pore volume (*V*_pore_) was estimated from the adsorption branch of the isotherm at *P*/*P*_0_ = 0.98. The microporous volume (*V*_micro_) was estimated from the t-plot. Pore volume distribution was estimated using the BJH model.

#### ICP-MS

Inductively coupled plasma mass spectroscopy (ICP-MS) was used to quantify the metal leaching of catalysts after the reaction. Post-reaction media were filtered to remove the catalyst and diluted in an acid (1% HNO_3_/0.5% HCl) aqueous solution to reduce the methanol content down to no more than 2%. Resulting solutions were analyzed using an Agilent 7900 ICP-MS instrument equipped with an I-AS autosampler. A blank was measured before the samples to carry out a blank subtraction if necessary. All samples were run in duplicate (minimum), and each value is an average of up to five independent measurements. Quantification was made against 5-point calibration plots using certified reference standards (Agilent). Samples and standards were analyzed along with an inline internal standard.

#### MP-AES

Microwave plasma-atomic emission spectroscopy (MP-AES) was performed using an Agilent 4100 MP-AES (Agilent Technologies) using the Agilent MP Expert software. Prior to the washing step of the catalyst preparation, the suspension was filtered and recovered, and 9 mL of the filtrate was acidified with 1 mL of aqua regia to ensure total metal dissolution prior to analysis. Signal response was recorded at two characteristic emission wavelengths for both Au (λ_1_ = 242.8 nm; λ_2_ = 267.6 nm) and Pd (λ_1_ = 340.5 nm; λ_2_ = 363.5 nm). Metal compositions were averaged and quantified against commercial calibration standards (Agilent; *r*^2^ > 0.999).

#### TEM/STEM

Transmission electron microscopy (TEM) was performed on a JEOL JEM-2100 operating at 200 kV. Energy dispersive X-ray analysis (EDX) was done using an Oxford Instruments X-MaxN 80 detector, and the data were analyzed using the Aztec software. Samples were prepared by dispersion in ethanol by sonication and deposited on 300-mesh copper grids coated with a holey carbon film. High angle annular dark-field (HAADF) scanning transmission electron microscopy (STEM) imaging was done using a JEOL ARM200CF operating at 200 kV.

#### XPS

X-ray photoelectron spectroscopy (XPS) analysis was performed on Thermo k-alpha^+^ spectrometer. Samples were pressed into wells of a copper sample plate using an isopropyl alcohol cleaned spatula and analyzed using microfocused monochromatic Al Kα radiation operating at 72 W (6 mA × 12 kV); pass energies of 40 and 150 eV were used for high-resolution and survey spectra, respectively, with corresponding step sizes of 0.1 and 1 eV. Charge compensation was performed using a combination of lower energy electrons and argon ions, with a background argon pressure of 10^–7^ mbar. Binding energies were calibrated using the C1s binding energy of carbon taken as 284.5 eV, typical for graphitic carbons.^30^ Data analysis was performed using CasaXPS after subtraction of a Shirley background using Scofield sensitivity factors and an electron escape dependence according to the TPP-2M formula.^31^

#### XRD

X-ray diffraction (XRD) analyses were conducted using a PANalytical X’pert Pro diffractometer with a Cu X-ray source operating at 40 kV and 40 mA. A Ge (111) single crystal monochromator was used to transmit selectively Cu Kα X-rays (λ = 0.154056 nm). Scans ranged 2θ from 5 to 80°. Diffractogram analyses were performed using the X’Pert High Score Plus software. Phase identification was carried out using the International Centre for Diffraction Data (ICDD).^32^

#### Zeta-Potential Determination

The ζ-potential analyses were performed using disposable folded capillary cells (DTS1070, Malvern Instruments) on a Zetasizer Nano ZS from Malvern Instruments Ltd., England. The ζ-potential values were calculated using Smulochowski’s model. Supports were suspended (100 ppm) in water, and colloidal suspensions were diluted in water (1:10). pH was adjusted to the desired value with concentrated sulfuric acid and monitored using a FiveEasy Standard pH Meter Line (Mettler Toledo) calibrated against buffer solutions. Analyses were run at least three times, and results correspond to the average of all three measurements.

### Catalyst Testing

#### Direct Synthesis of Hydrogen Peroxide

Catalytic activity toward the direct synthesis of hydrogen peroxide was evaluated using a Parr Instruments stainless steel autoclave with a nominal volume of 0.1 L and a maximum working pressure of 14 MPa. Reaction parameters have previously been demonstrated to be optimum for the direct synthesis of hydrogen peroxide.^33,34^ The autoclave was charged with the catalyst (0.01 g) and solvents (2.9 g H_2_O and 5.6 g MeOH, both HPLC grade, Fisher Scientific) and then purged three times with 5% H_2_/CO_2_ (0.7 MPa) before filling with 5% H_2_/CO_2_ (2.9 MPa) and 25% O_2_/CO_2_ (1.1 MPa). The reactor temperature was decreased to 2 °C, and then the mixture was stirred (1200 rpm) for 0.5 h. H_2_O_2_ productivity (mol_H2O2_ kg_cat_^–1^ h^–1^) was determined by titrating aliquots (*ca.* 0.5 g) of the final filtrated solution after use in the direct synthesis reaction with acidified Ce(SO_4_)_2_ (8.5 mmol L^–1^) in the presence of the ferroin indicator. H_2_O_2_ productivity (mol_H2O2_ mmol^–1^_metal_ h^–1^) was normalized with respect to the actual metal loading determined by MP-AES. For the *in situ* acid addition experiments, the amount of acid (5.4 × 10^–4^ g of 2 wt % H_2_SO_4_ aqueous solution) added into the liner was equal to that required to prepare 0.01 g of the catalyst, which corresponds to the catalyst mass present in a standard H_2_O_2_ synthesis experiment when using a 1wt % AuPd heterogeneous catalyst. For experiments using a colloidal suspension, the amount of water was reduced to obtain a final metal concentration (Au + Pd) of 17.24 ppm, equal to that present in a standard H_2_O_2_ synthesis experiment when using a 1 wt % AuPd supported heterogeneous catalyst.

Catalytic conversion of H_2_ and selectivity toward H_2_O_2_ were determined by gas chromatography using a Varian 3800 GC equipped with a Porapak Q column and a TCD. H_2_ conversion (eq 1) and H_2_O_2_ selectivity (eq 2) were calculated as follows:
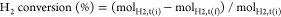
1
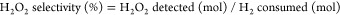
2

#### Hydrogen Peroxide Degradation

Catalytic activity toward H_2_O_2_ degradation was determined in a similar way to the direct synthesis activity of a catalyst. The autoclave was charged with the catalyst (0.01 g), MeOH (5.6 g, HPLC grade, Fisher Scientific), H_2_O_2_ (50 wt %, 0.69 g, Merck), and water (2.21 g, HPLC grade, Fisher Scientific). Prior to the addition of the catalyst to the reaction solution, three aliquots (0.05 g) were removed from the solution and titrated with acidified Ce(SO_4_) (0.01 mol L^–1^) in the presence of the ferroin indicator to determine the exact H_2_O_2_ initial concentration. The autoclave was pressurized with 2.9 MPa 5% H_2_/CO_2_, cooled down to 2 °C, and then stirred for 0.5 h (1200 rpm). H_2_O_2_ degradation activity (mol_H2O2_ kg_cat_^–1^ h^–1^) was determined by titrating aliquots (*ca.* 0.05 g) of the final filtrated solution after the reaction with acidified Ce(SO_4_)_2_ (8.5 mmol L^–1^) in the presence of the ferroin indicator. For the *in situ* acid addition tests, 5.4 × 10^–4^ g of acid (H_2_SO_4_, 98 wt %) was added as a 2 wt % aqueous solution. H_2_O_2_ degradation activity (mol_H2O2_ mmol^–1^_metal_ h^–1^) was normalized with respect to the actual metal loading determined by MP-AES.

#### Catalyst Reusability

A similar procedure to that outlined above for the direct synthesis of H_2_O_2_ is followed to determine catalyst reusability for the direct synthesis of H_2_O_2_ and its subsequent degradation. Following the initial synthesis test (performed with 0.05 g of the catalyst), the catalyst is recovered by filtration and dried in a vacuum oven (50 °C, <0.005 MPa, static air). A total of 0.01 g of the recovered sample was used to conduct the second synthesis and degradation tests.

## Results and Discussion

**Figure 1:**
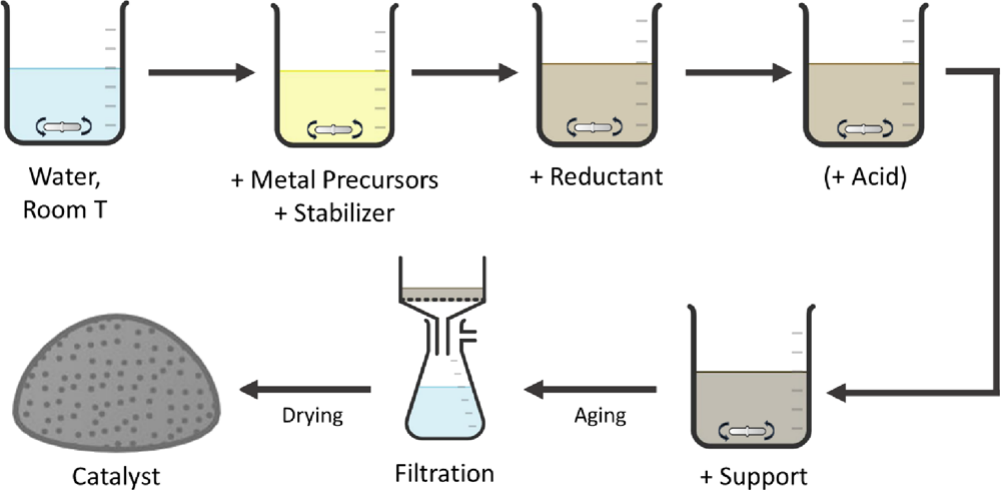
Catalyst preparation via the sol immobilization method.

It is generally considered that the addition of acid during the sol immobilization preparation results in increased metal deposition on the catalyst support.^35,36^ Full metal immobilization can however be easily achieved by working under stabilizer-free conditions without any need of acid, which leaves ambiguity to the precise purpose of the acid in the sol immobilization preparation. To fully understand the role acid addition plays in the sol immobilization preparation, illustrated in Figure 1, we studied the effect of adding acid to the colloidal suspension during the catalyst preparation on the activity of AuPd/C catalysts for the direct synthesis reaction. We prepared 1 wt % AuPd catalysts supported on commercially available carbon (GNP or KBB), using polyacrylic acid (PAA) as the stabilizing agent, in the presence or absence of acid (H_2_SO_4_, 133 ppm). The pH of the solution (containing the reductant, water, and metal precursors) dropped from 3.05 to 2.74 on addition of acid. The carbon support was then added. The resulting catalyst was filtered, washed, and dried prior to the evaluation of catalytic performance toward the direct synthesis of hydrogen peroxide and its subsequent degradation (Table 1).

**Table 1:** Catalytic Testing Results for PAA-Stabilized AuPd/C Catalysts toward the Direct Synthesis of Hydrogen Peroxide and Its Subsequent Degradation

catalyst	acid addition	H_2_O_2_ productivity^a^ (mol_H2O2_ kg^–1^_cat_ h^–1^)	H_2_O_2_ degradation^b^ (mol_H2O2_ kg^–1^_cat_ h^–1^)	H_2_O_2_ selectivity (%)
1%AuPd/GNP	no	62	55	14
1%AuPd/GNP-H^+^	yes	202	460	38
1%AuPd/GNP (H^+^*in situ)*	*in situ*	40	67	17
1%AuPd/KBB	no	17	57	2
1%AuPd/KBB-H^+^	yes	46	529	25
1%AuPd/KBB (H^+^*in situ)*	*in situ*	4	8	3

a H_2_O_2_ direct synthesis reaction conditions: catalyst (0.01 g), H_2_O (2.9 g), MeOH (5.6 g), 5%H_2_/CO_2_ (2.9 MPa), 25% O_2_/CO_2_ (1.2 MPa), 0.5 h, 2 °C, 1200 rpm.

b H_2_O_2_ degradation reaction conditions: catalyst (0.01 g), H_2_O_2_ (50 wt % 0.68 g), H_2_O (2.22 g), MeOH (5.6 g), 5% H_2_/CO_2_ (2.9 MPa), 0.5 h, 2 °C, 1200 rpm.

Bare supports are only active toward the degradation of H_2_O_2_ (55 and 95 mol_H2O2_ kg^–1^_cat_ h^–1^, for GNP and KBB, respectively), which is consistent with the literature.^13,37,38^ The addition of the acid (H_2_SO_4_, 133 ppm) during the catalyst preparation (denoted as 1%AuPd/Carbon-H^+^) is observed to result in a drastic increase in catalytic activity and selectivity toward the direct synthesis of H_2_O_2_ compared to catalysts prepared in the absence of acid (denoted as 1%AuPd/Carbon) for both the KBB and GNP materials.

Acids are often used as additives during liquid phase hydrogen peroxide synthesis from H_2_ and O_2_,^8,39^ with a general acceptance that acids stabilize hydrogen peroxide by hindering the degradation pathways. As a result, both apparent catalytic productivity and selectivity toward H_2_O_2_ are enhanced.^8,10,39,40^ More recently, Wilson and Flaherty showed that protons, added to the reaction medium from mineral acids, could be directly involved in the production of H_2_O_2_ by facilitating the reduction of O_2_ at the surface of supported Pd clusters.^11^ The authors underline the importance of the acid counterion for the determination of the H_2_O_2_ selectivity through electronic modifications of the solvent–Pd interface and obstruction of the sites responsible for the irreversible cleavage of the O–O bond, systematically leading to the formation of water.^11^ However, no change in the pH of the reaction solution was observed when our 1%AuPd/Carbon-H^+^ catalysts were added to the reaction solvent compared to the non-acid homologues and the supports. This observation is likely due to the washing step during the catalyst preparation and suggests no leaching of protons and/or counterions (SO_4_^2–^) from the catalysts prepared with acid in the reaction solvent. Moreover, adding acid (H_2_SO_4_, 133 ppm) directly into the reaction medium (Table 1, ″H^+^*in situ*″) does not lead to any enhancement of the catalytic activity of non-acid-prepared catalysts. These results suggest that the beneficial role of adding acid to the catalyst preparation on the catalytic performances does not occur through the promotional role of protons (and their counterions, in this case, SO_4_^2–^) in the reaction medium under these conditions.

### Increased NP Immobilization Fraction

During the preparation, the color of the filtrate varied depending on whether acid was added, with no color noted in the presence of acid. Without acid, the filtrate had a dark/brown color, indicating the incomplete adsorption of the PAA-stabilized metal nanoparticles onto the support.^41^ Elemental analyses of the filtrates allow for the determination of the immobilized fraction (I.F.) of metals onto the support by deducting the amount of metal detected from the measured amount present at the start (Table 2).

**Table 2:** MP-AES-Derived (Au, Pd) Immobilized Fraction of 1%AuPd/C Catalysts Prepared by Sol Immobilization with or without Acid Addition

	immobilized fraction (%)	
catalyst	Au	Pd
1%AuPd/GNP	49	46
1%AuPd/GNP-H^+^	100	100
1%AuPd/KBB	22	19
1%AuPd/KBB-H^+^	100	100

For the two different carbon-based supports used, similar amounts of both Au and Pd are immobilized in the absence of acid, indicating no metal-dependent metal deposition. In the absence of acid, only a small fraction of the metal (20–50%) was immobilized onto the supports. Adding acid to the preparation mixture (H_2_SO_4_, 133 ppm) allows for the complete adsorption of the suspended nanoparticles onto both supports.

#### Electrostatic Interactions

In solutions, the suspended nanoparticles (NPs) consist of metallic species enclosed in the stabilizer envelope.^42,43^ As such, the physicochemical properties of the stabilizer will dictate the overall interactions of the nanoparticles with their surroundings. The measure of the intensity of the electrostatic interactions between nanoparticles is called the zeta potential, which can be defined as the electrical potential difference between the slipping plane of a particle and the solvent. The zeta potential of both supports and the PAA-stabilized nanoparticles was measured in the solution and summarized in Table 3.

**Table 3:**
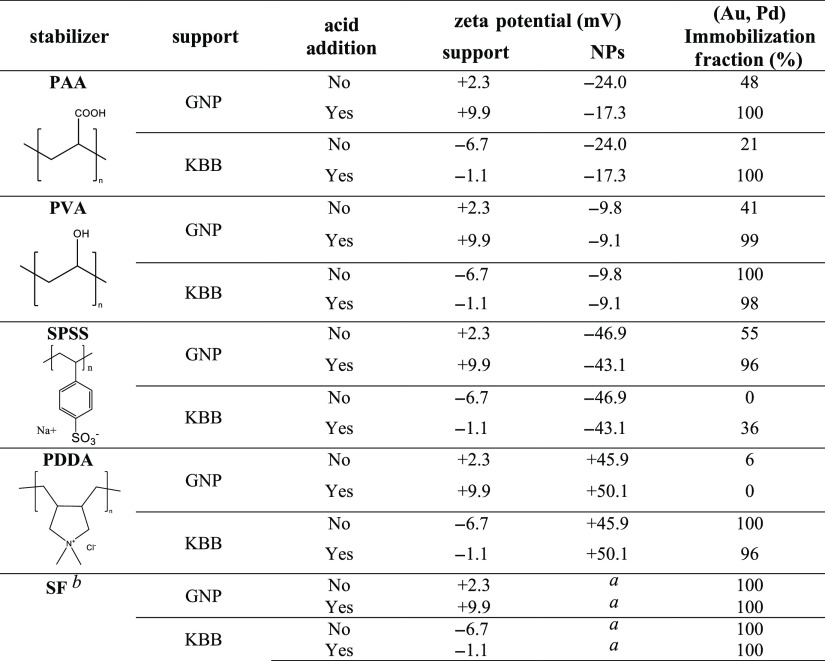
(Au, Pd) Immobilization Fraction for Each Stabilizer-Support System and Their Zeta Potential, Depending on the Acid Addition (H_2_SO_4_, 133 ppm)

a Suspension not stable enough to allow analysis.

b SF: stabilizer-free.

Without acid, suspended GNP has a slightly positive charge (+2.3 mV), while both suspended KBB and the unsupported PAA-stabilized nanoparticles are charged negatively (−6.7 and −24.0 mV, respectively). Acid addition increases the zeta potential of all adsorption components (supports and suspended nanoparticles). Upon acid addition, GNP becomes more positive (+9.9 mV), whereas the zeta potential of KBB tends to neutralize (−1.1 mV). This increase is likely due to the protonation and/or the neutralization of functional groups at the surface of the supports, as described later. In the case of PAA-stabilized nanoparticles, the increase (from −24.0 to −17.3 mV) can be explained by the partial neutralization of the carboxyl groups of the anionic polymer by the added protons.^44^

Coulomb’s law states that the amplitude of the electrostatic forces between two bodies is directly proportional to the product of their charges.^45^ Adding acid modifies these amplitudes and can switch the nature of the electrostatic system (repulsive, neutral, or attractive) between the suspended NPs and the support.

In the absence of acid, the zeta potential of GNP is close to the neutral potential (+2.3 mV) and can be considered as approximately neutral.^46^ Therefore, electrostatic interactions are thought to play a negligible role in the adsorption process, which is then governed by hydrogen bonds.^47,48^ When acid is added, the zeta potential of GNP increases (+9.9 mV) and attractive electrostatic interactions take place with the suspended nanoparticles. Adding acid results in a transition from a non-electrostatic system (I.F._PAA-GNP_ = 48%) to an attractive electrostatic system, which will favor the adsorption of the suspended nanoparticles (I.F._PAA-GNP-H+_ = 100%).

Repulsive electrostatic interactions occur between KBB (−6.7 mV) and the stabilized NPs (−24.0 mV) when no acid is added. Adding acid will increase the zeta potential of the support to near 0 mV (−1.1 mV), resulting in negligible electrostatic interactions between the support and the suspended NPs. Here, the acid addition allows moving from an unfavorable repulsive system to a neutral one, leading to an increased adsorption fraction (I.F._PAA-KBB_ = 21%; I.F._PAA-KBB-H+_ = 100%).

In the absence of electrostatic interactions, hydrogen bonds are thought to be the main force for the adsorption of PAA onto the adsorbent.^49^ Upon decreasing pH, PAA (p*K*_a_ = 5.98) becomes less ionized and neutralized carboxyl groups can undergo hydrogen bonding with the support.^44^ The difference in both H-bonding capacities of our supports could explain the different nanoparticle adsorption fractions when electrostatic interactions are negligible (I.F._PAA-GNP_ = 48%; I.F._PAA-KBB-H+_ = 100%).

**Figure 2:**
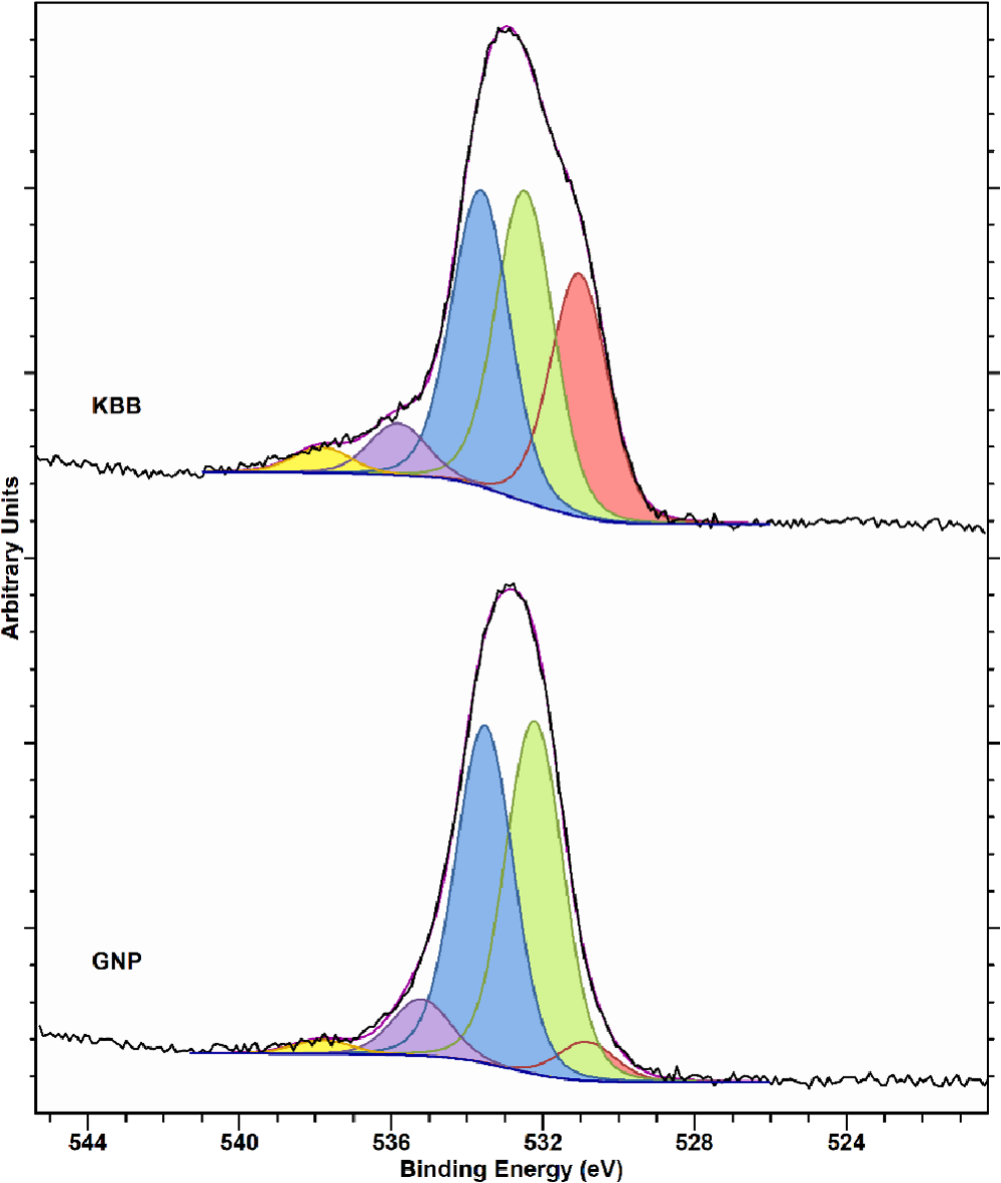
O(1s) spectra of both pristine supports. KBB (top) and GNP (bottom).

The difference in the electrostatic behavior of our chosen supports can be explained by comparing their surface chemistry. Both supports have a similar XPS-derived surface oxygen content (GNP: 7.0%; KBB: 9.0%), with the O(1s) spectra of both pristine materials depicted in Figure 2. The O(1s) signal of KBB consists of three main components. The first centered at 531.1 eV can be assigned to C=O species, such as carboxyl groups. The second peak at 532.5 eV can be assigned to the single-bonded oxygen in O–C=O and C–O containing functions such as phenols, alcohols, ester-type linkages, and isolated carbonyl functions. A third significant peak is found at 533.7 eV and characteristic of organic carbonate functions such as that found in poly(bisphenol A carbonate) and similar compounds, while the smaller peaks between *ca.* 535 and 538 eV are related to the shake-up satellite structure of carboxyl-containing functions.^30,31,50^ In the case of GNP, the fitting of the O(1s) spectrum yields similar peaks but in clearly different concentrations to those in KBB. Of note is the lower intensity of the higher binding energy satellite peaks indicating a significantly different distribution of carbonyl-containing functions to that in KBB.

We further investigated the oxygen-containing functional groups at the surface of our supports using Boehm’s titration method (Table 4).^29^ Most oxygen-containing groups at the GNP surface are lactone, with low carboxyl and phenol contents. Compared to GNP, KBB has a higher concentration of carboxylic groups at its surface, whereas lactone and phenol contents are similar, consistent with XPS results.

**Table 4:** Oxygen-Containing Carbon Surface Functional Groups

support	carboxylic (μmol g^–1^)	lactonic (μmol g^–1^)	phenolic^a^ (μmol g^–1^)
GNP	4 ± 21	449 ± 65	93 ± 66
KBB	150 ± 24	372 ± 56	321 ± 78

a Phenolic groups and hydroxyl functions with similar p*K*_a_ are detected.

Based on the fitted O(1s) spectra of GCN, one could have expected an equal concentration of phenolic and lactonic groups at the carbon surface, with minimal carboxyl content. Yet, most oxygen-containing groups react with Na_2_CO_3_ during the titration protocol and are therefore labeled as lactones in Boehm’s system. However, the p*K*_a_ values of the functional groups strongly depend on their environment and a generic allocation of the groups can be delicate.^51,52^ In our case, it is likely that part of phenol groups has reacted with Na_2_CO_3_, resulting in a bias during the quantification of both lactone and phenol groups.

At higher pH (without acid), most carboxyl groups at the KBB surface will be deprotonated, leading to a negative charge and hence a negative zeta potential (−6.7 mV). Decreasing the pH will progressively neutralize these groups, leading to a neutral potential (−1.1 mV). Conversely, GNP does not have carboxyl groups but lactones and phenols. At higher pH, such groups are neutral from an electrostatic point of view (+2.3 mV). Adding acid will protonate them, leading to a positive charge and hence a positive zeta potential (+9.9 mV).

**Figure 3:**
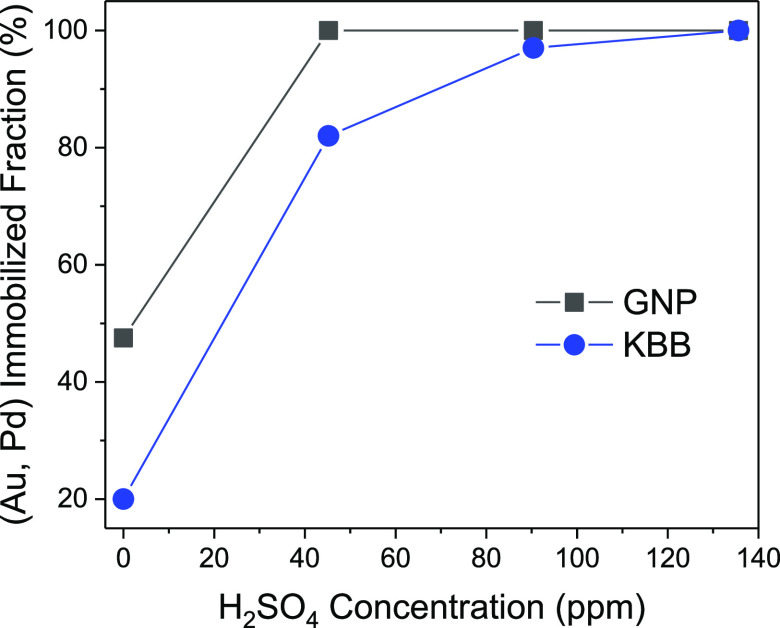
(Au, Pd) immobilized fraction onto both GNP and KBB as a function of the amount of acid added to the suspension. The immobilized fraction was determined from MP-AES analysis of the preparation filtrate.

Increasing the amount of acid added during the preparation increases the adsorption fraction of the suspended nanoparticles onto the support (Figure 3). The presence of only 43 ppm of H_2_SO_4_ (98 wt %) is enough to immobilize 100% of the nanoparticles onto GNP, suggesting that the added protons give sufficient charge to the support to switch from the neutral electrostatic system (without acid added) to the attractive one. KBB requires a larger amount of acid (133 ppm) to reach a 100% immobilization fraction. This can be explained by the progressive neutralization of the carboxyl groups, which needs to be almost complete to overcome the repulsive electrostatic interactions with the negatively charged nanoparticles. A further increase in acid concentration (up to *ca.* 8500 ppm; pH = 1) does not modify the adsorption fraction (100%). Additional protons will increase the zeta potential of both the adsorbent (support) and the adsorbate (suspended nanoparticles). Upon acid addition, the zeta potential of the suspended nanoparticles will tend to 0 due to the neutralization of the carboxyl groups of the PAA, while the zeta potential of GNP will continue to increase through the protonation of its surface. At some point, the zeta potential of KBB will be reversed to a positive value *via* the simultaneous neutralization of the carboxyl groups and the progressive protonation of the lactones/phenols. This will lead to an attractive electrostatic system with the suspended nanoparticles. The catalytic activity of the materials prepared upon increasing H_2_SO_4_ concentration follows the same trend as the metal immobilized fraction (see Table S1). A sharp activity increase is observed at low acid concentrations before reaching a plateau around 100 ppm.

**Figure 4:**
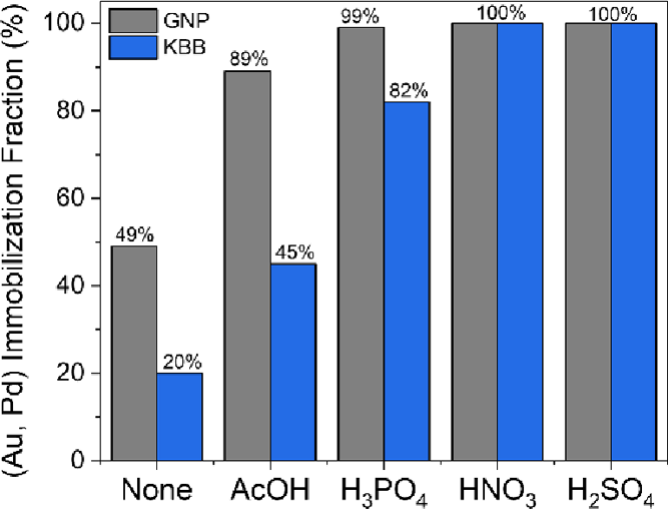
(Au, Pd) immobilized fraction onto both GNP and KBB as a function of the nature of the acid added. The immobilized fraction was determined from MP-AES analysis of the preparation filtrate.

For a given concentration of acid, changing the strength of the acid modifies the NPs’ immobilization fraction: the weaker the acid, the lower the immobilization fraction (Figure 4). Using a weak acid (acetic acid, p*K*_a_ = 4.8) allows for the partial immobilization of the nanoparticles onto both supports (89% for GNP; 45% for KBB). Using a stronger acid (H_3_PO_4_; p*K*_a1_ = 2.1, p*K*_a2_ = 7.2, and p*K*_a3_ = 12.3), immobilization fractions of 99 and 82% were reached for GNP and KBB, respectively. Adding a strong acid (HNO_3_ (p*K*_a_ = −1.4) or H_2_SO_4_ (p*K*_a_ = −3.0)) results in the complete immobilization of the nanoparticles onto both supports. For a given metal immobilization fraction, the catalytic activity does not depend on the nature of the acid (Table S2). For example, 1%AuPd/GNP prepared with H_2_SO_4_ has a similar H_2_O_2_ productivity compared to the analogue prepared with HNO_3_ (202 vs 191 mol_H2O2_ kg^–1^_cat_ h^–1^, respectively) and both catalysts exhibit metal immobilization fractions of 100%.

These results corroborate our previous observations. Adding acid modifies the adsorption fractions through modifications of the electrostatic interactions between the suspended nanoparticles and the support. The stronger the acid and/or the higher the acid concentration, the bigger these modifications.

In the case of PAA-stabilized NPs, the fact that the new electrostatic system is more favorable from an adsorption point of view is due to the anionic nature of the stabilizer, which gives a negative zeta potential to the NPs regardless of the acid addition. We extended our studies on the importance of the electrostatic interactions for the adsorption of the suspended NPs onto carbons by preparing a series of 1wt % AuPd catalysts supported on GNP and KBB, using a range of stabilizers, using H_2_SO_4_ (133 ppm) as the acid. Metal immobilization fractions and zeta potential of each system are summarized in Table 3.

The nature of the stabilizer has a strong influence on the metal immobilization fractions. Changing the stabilizer results in changing the zeta potential of the suspended nanoparticles, hence modifying the electrostatic interactions between the suspended nanoparticles and the support. The effect of adding acid (H_2_SO_4_, 133 ppm) to the preparation on the metal immobilization fractions depends on the stabilizer-support system used. Stabilizers with a strong ionic character (i.e., SPSS or PDDA) will lead to suspended nanoparticles with a zeta potential of high amplitude. Such nanoparticles undergo strong electrostatic interactions with the support, whose nature (repulsive or attractive) depends on the zeta potential of the support. In such systems, adding acid (H_2_SO_4_, 133 ppm) has little effect on the metal immobilization fraction as the resulting changes of zeta potential of both support and suspended nanoparticles are negligible compared to the high initial zeta potential of the suspended nanoparticles. The effect of adding acid (H_2_SO_4_, 133 ppm) to the preparation on the metal immobilization fractions is steeper when using a stabilizer with a slight ionic character (i.e., PAA or PVA). The variations of zeta potential of both suspended nanoparticles and support, due to the acid addition, are high enough to trigger a switch of the nature of the electrostatic system.

Full immobilization of the metal nanoparticles is observed when the preparation is performed without a stabilizer (SF, stabilizer free), regardless of the support or the acid addition. Without a stabilizer, the chemistry of the preparation is closer to a deposition–precipitation technique than a sol immobilization method, resulting in different driving forces for the metal adsorption.

#### Diffusion Limitations

**Figure 5:**
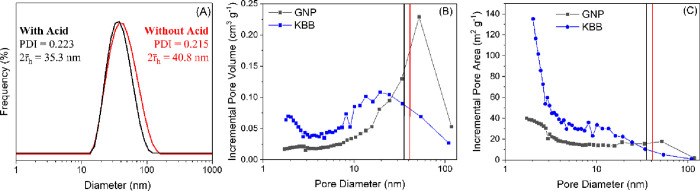
(A) Particle size distribution by frequency of PAA-stabilized AuPd nanoparticles, with or without acid. (B) Incremental pore volume. (C) Incremental surface area of GNP (blue) and KBB (gray). Vertical lines represent the size of the PAA-protected nanoparticles determined by DLS. Black line = with acid (H_2_SO_4_, 133 ppm); red line = without acid.

Beyond electrostatic interactions, adding acid could increase nanoparticle immobilization fractions through improved metal distribution, including into the pore structure of the support, leading to the larger availability of adsorption sites. Upon decreasing pH during catalyst preparation, the ionization degree of PAA decreases and repulsive intramolecular electrostatic forces are reduced. As a result, a conformation change occurs: the polymer goes from an extended to a shorter, coiled structure.^44,47,53,54^ This change of conformation occurs around pH = 6 in water and depends on the ionic strength of the solvent.^44^ Dynamic light scattering (DLS) analyses of our colloids reveal that adding acid does not trigger the conformation change, with PAA likely in the coiled structure. The size of the suspended nanoparticles does not vary significantly upon acid addition (35.3 vs 40.8 nm) (Figure 5).

Textural properties of the supports are summarized in Table 5. Both supports depict a broad pore size distribution (Figure 5). The high surface area of the supports is mainly due to the presence of micro- and mesopores, whose diameter is smaller (or similar) than the diameter of the suspended nanoparticles. Therefore, only a small fraction of the porosity of each support is available for nanoparticles to diffuse in it. This fraction, as well as the corresponding pore area, is not thought to be significant compared to the external area. Therefore, the role of acid on the diffusion limitations can be considered negligible.

**Table 5:** Textural Properties of the Supports

support	*A*_sp, BET_ (m^2^ g^–1^)	*V*_pore_ (cm^3^ g^–1^)
GNP	893	1.1
KBB	1693	2.1

#### Immobilization Time

Immobilization time can vary significantly from one study to another. While most studies opt for an immobilization time ranging from 0.5 to 2 h, some works report aging durations up to several days.^55−57^ This increase is often justified to enhance the extent of metal immobilized. However, despite longer aging times, full metal immobilization is not guaranteed and is not always reported.^57^

**Figure 6:**
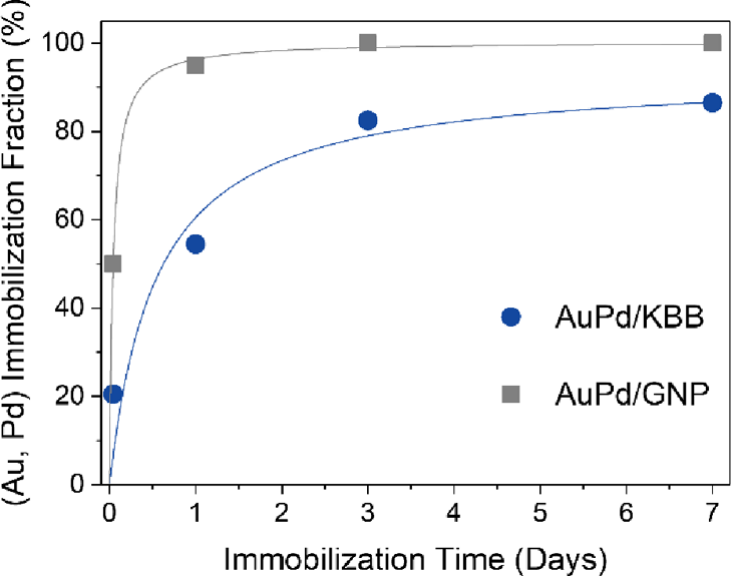
(Au, Pd) Immobilization fraction as a function of time.

We investigated the influence of the immobilization time of the PAA-protected colloidal suspension on the immobilization fraction for both supports in the absence of acid (Figure 6). The immobilized fraction increases rapidly before reaching a plateau. For 1%AuPd/GNP, 1 day of aging is enough to immobilize 95% of the nanoparticles and full immobilization is observed within 3 days. For 1%AuPd/KBB, the increase is slower. After 7 days, only 87% of the nanoparticles are immobilized. These results contrast strongly with the full immobilization of the nanoparticles reached within 30 min of aging when acid is added.

The difference in immobilization fraction over time between both supports can, in part, be explained by their different textural properties. Although KBB has a higher surface area than GNP (Table 5), most of it corresponds to the walls of micro- and mesopores (Figure 5). These pores are too small to allow the diffusion of the protected nanoparticles. As a result, the corresponding surface area is not available for adsorption. Conversely, GNP has larger pores, in which the protected nanoparticles can diffuse. The surface area of these larger pores acts as the adsorption site, leading to a higher immobilization fraction.

Alternatively, Comotti and co-workers observed that the immobilization time required to fully immobilize PVA-protected Au nanoparticles on a range of activated carbons differed depending on the support used. The catalyst requiring the longest immobilization time depicted larger nanoparticles, suggesting that, upon increasing immobilization time, PVA-protected Au nanoparticles tend to agglomerate before precipitating onto the support surface.^58^ However, this is unlikely in the case of PAA-protected nanoparticles because of the bigger electrostatic repulsion between the suspended nanoparticles, as evidenced by their lower zeta potential (Table 3) and their similar size over time (Figure S1).^18^

Therefore, it can be concluded that adding acid increases the adsorption kinetics and the adsorption equilibrium through the modification of both the amplitude and the nature of the electrostatic interactions taking place between the suspended nanoparticles and the support. The more acid added (and/or the stronger the acid), the bigger these modifications, with the nature of both the stabilizer and the support dictating the initial electrostatic system.

### Beyond Actual Metal Loading

Normalizing the catalytic activity with respect to the actual metal loading reveals that the promotional effect of the acid addition goes beyond the increased metal immobilization fraction. Catalysts prepared with acid are more active and more selective toward the synthesis of H_2_O_2_ than their analogues prepared in the absence of acid (Table 6).

**Table 6:** Catalytic Testing Results for PAA-Stabilized AuPd/C Catalysts toward the Direct Synthesis of Hydrogen Peroxide and Its Subsequent Degradation

catalyst	actual metal loading (wt %)	H_2_O_2_ productivity^a^ (mol_H2O2_ mmol^–1^_metal_ h^–1^)	H_2_O_2_ degradation^b^ (mol_H2O2_ mmol^–1^_metal_ h^–1^)	H_2_O_2_ selectivity (%)
1%AuPd/GNP	0.5	1.9	1.7	14
1%AuPd/GNP-H^+^	1	2.8	6.4	38
0.5%AuPd/GNP-H^+^	0.5	2.6	6.1	35
1%AuPd/KBB	0.2	1.2	3.3	2
1%AuPd/KBB-H^+^	1	1.9	7.3	25
0.2%AuPd/KBB-H^+^	0.2	1.8	7.0	25

a H_2_O_2_ direct synthesis reaction conditions: catalyst (0.01 g), H_2_O (2.9 g), MeOH (5.6 g), 5%H_2_/CO_2_ (2.9 MPa), 25%O_2_/CO_2_ (1.2 MPa), 0.5 h, 2 °C, 1200 rpm.

b H_2_O_2_ degradation reaction conditions: catalyst (0.01 g), H_2_O_2_ (50 wt % 0.68 g), H_2_O (2.22 g), MeOH (5.6 g), 5% H_2_/CO_2_ (2.9 MPa), 0.5 h, 2 °C, 1200 rpm.

We subsequently prepared two catalysts (0.5%AuPd/GNP-H^+^ and 0.2%AuPd/KBB-H^+^) with a nominal metal loading equal to that obtained in the absence of acid. During the preparation of these catalysts, acid was added to ensure the full immobilization of the metals, confirmed by analysis of the catalyst preparation filtrate by MP-AES. To ensure a relevant comparison with the original protocol, amounts of both stabilizer and reducing agent were adjusted to keep the same reagent ratios (NaBH_4_/metals = 5 mol/mol; PAA/metals = 1.15 wt/wt). Such prepared catalysts have similar normalized activity and selectivity than their analogues with a higher loading (Table 6). The results evidence that adding acid (H_2_SO_4_, 133 ppm) improves catalytic performances beyond that expected from increased metal loading, likely due to the modification of the nanoparticles during the preparation.

Both the composition and structure of the active nanoparticles are key to obtaining high activity and selectivity for the direct synthesis of hydrogen peroxide.^8^ Pure Pd supported nanoparticles are highly active toward the direct synthesis of hydrogen peroxide but typically suffer from poor selectivity, with high H_2_O_2_ degradation rates.^8^ Alloying Au with Pd results in a drastic increase in both activity and selectivity. The synergy is only observed when metals are alloyed, and phase segregation does not lead to any catalytic improvement.^12^ Testing monometallic catalysts supported on both our supports corroborates the literature (Table S3). The nature of the Au–Pd synergy is thought to be the result of electronic, structural, and isolation effects.^8,59^ DTF studies of Pd and AuPd surfaces indicate that the role of the Au in an AuPd (111) surface is to allow the facile desorption of H_2_O_2_ (preventing overhydrogenation to water) and to suppress O–O cleavage, resulting in increased H_2_O_2_ selectivity/reduction in H_2_O_2_ degradation.^59^

**Figure 7:**
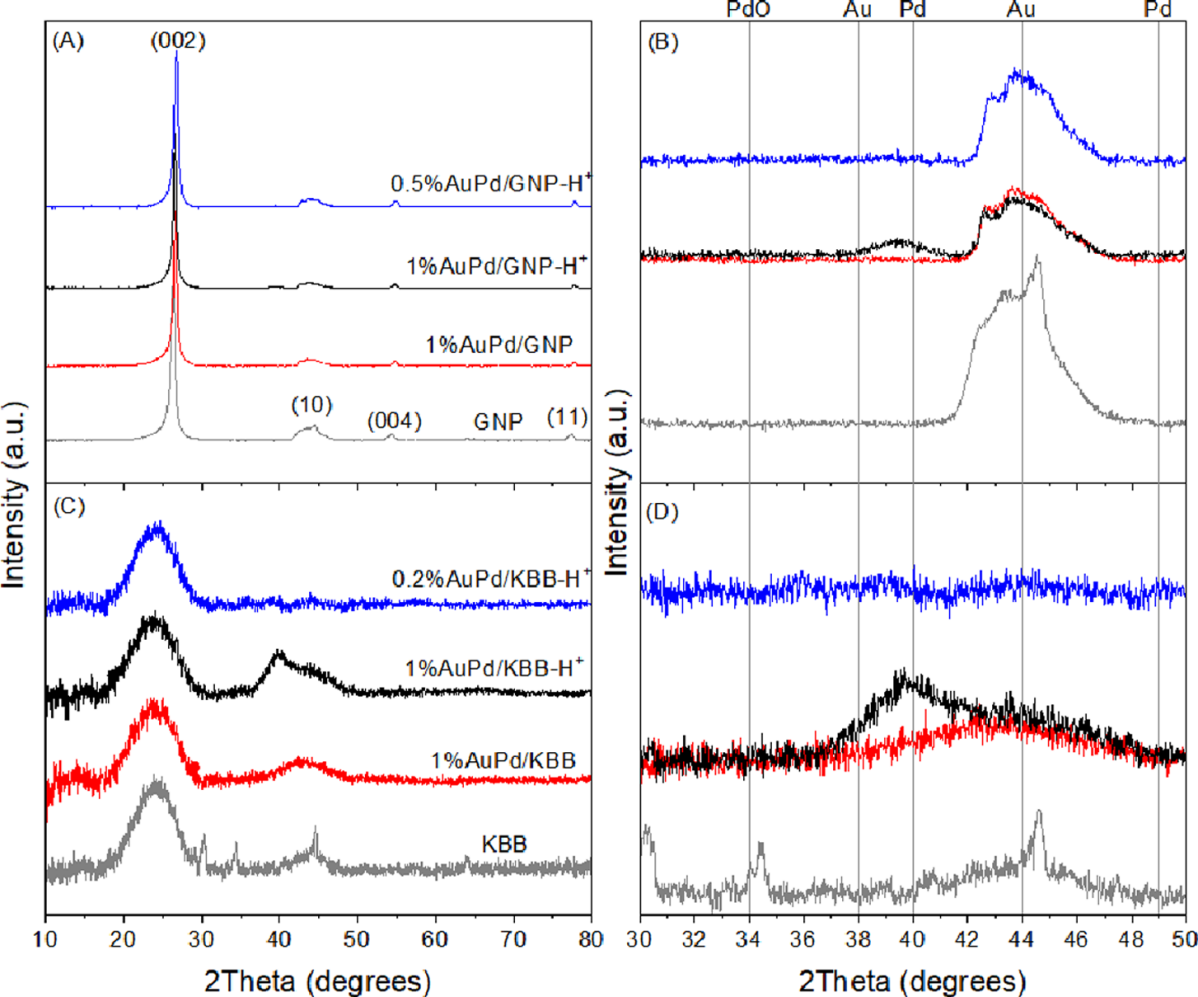
Diffractograms (A, C) and magnification (B, D) of pristine supports (gray) and 1%AuPd/C catalysts prepared using PAA as the stabilizer, with (black) or without (red) acid. Diffractograms of 0.5%AuPd/GNP-H^+^ and 0.2%AuPd/KBB-H^+^ are depicted in blue.

X-ray diffractograms of our samples are depicted in Figure 7. Main diffraction reflections occur at 2θ = 26, 42, 45, 50, 55, and 78° and correspond to graphite planes (002), (100), (101), (102), (004), and (110), respectively. In the case of pristine KBB, diffraction peaks can be spotted at 2θ = 30, 34, 35, and 45°. These peaks correspond to a Na_3_PO_4_ phase, which corroborate with XPS results, revealing the presence of both P and Na at the surface of the pristine support. No peak related to a Na_3_PO_4_ phase is observed for loaded catalysts, which can be explained by the dissolution of the salt during the preparation. No peak related to Au (2θ = 38, 44, and 70°), Pd (2θ = 40, 49, and 67°), or PdO (2θ = 34°) is observed, indicating no phase segregation. Instead, a broad diffraction peak appears at 2θ = 39° (between the expected Au and Pd reflections) in catalysts prepared with acid, corresponding to an AuPd alloy phase. This peak is less discernible for catalysts prepared without acid due to their lower metal loading, as evidenced by the absence of a diffraction signal for metal-free catalysts (Figure S2), as well as 0.5%AuPd/GNP-H^+^ and 0.2%AuPd/KBB-H^+^. These results indicate a good metal dispersion with no obvious segregation of Au or Pd and suggest that nanoparticles consist of a random AuPd alloy.^60,61^

**Figure 8:**
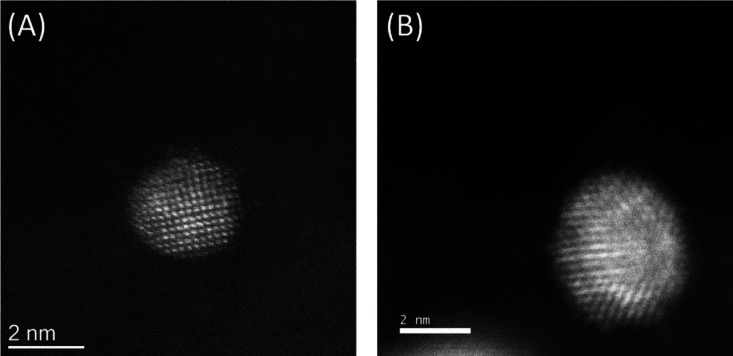
STEM-HAADF images of (A) 1%AuPd/GNP and (B) 1%AuPd/GNP-H^+^.

STEM-HAADF analyses (Figure 8) of both 1%AuPd/GNP and 1%AuPd/GNP-H^+^ confirm these observations; regardless of the acid addition, nanoparticles are randomly alloyed with no phase segregation. The HAADF-STEM images of individual particles in Figure 8 show varying contrasts among individual atomic columns, indicating the mixing of heavier element Au (*Z* = 79) and Pd (*Z* = 46) in these particles and consistent with previous studies on AuPd/carbon catalysts.^61^

.

XPS analyses of both catalysts show that all metals are in the metallic state regardless of the acid addition. This is in agreement with the literature for AuPd/C catalysts prepared by sol immobilization using NaBH_4_ (NaBH_4_/metal = 5 mol/mol) as the reducing agent^16,62,63^ and dismisses the hypothesis of a potential Au-core PdO-shell morphology, often reported as a key factor for the enhancement of the catalytic performances of AuPd nanoparticles supported on oxide supports.^8^

Tian *et al.* showed that small Pd nanoclusters (1.4–2.5 nm) supported on hydroxyapatite were particularly appropriate for the direct synthesis of hydrogen peroxide, with H_2_O_2_ selectivity reaching up to 94%, and suggested the presence of Pd^δ+^ as a key factor for such catalytic performances.^64^ If the presence of such nanoclusters at the surface of our catalysts cannot be ruled out, it is unlikely that the acid addition favors their formation since the XPS-derived Au/Pd ratios increase upon acid addition (Table 7). Moreover, no shift in the Pd XPS signal is observed for acid-prepared catalysts compared to their analogues prepared without acid (Figure S3).

**Table 7:** Au/Pd XPS-Derived Molar Ratio and Median and Mean Size of 1%AuPd/C Catalysts Prepared Using PAA as the Stabilizer with or without Acid

catalyst	Au/Pd molar ratio (−)	median size (nm)	mean size (nm)
1%AuPd/GNP	0.2	6.1	6.5 ± 2.7
1%AuPd/GNP-H^+^	0.8	4.4	4.5 ± 1.4
1%AuPd/KBB	1.1	5.8	6.2 ± 1.9
1%AuPd/KBB-H^+^	1.5	4.2	4.3 ± 1.3

Pritchard and co-workers demonstrated that the actual composition of AuPd nanoparticles on carbon (Aldrich G60) prepared by a similar sol immobilization varies depending on the size of the nanoparticles. Using XEDS, they showed that smaller nanoparticles (2–3 nm) were Au-rich, medium-sized nanoparticles (5–7 nm) were made of similar amounts of both Au and Pd, and larger nanoparticles (10–12 nm) were Pd-rich.^61^ The difference in the reduction rate of both metals is thought to be the reason for such variations.^65^

Taking the above into consideration, the increase in the Au/Pd XPS-derived atomic ratio upon acid addition could be explained by an increased metal dispersion: the higher the dispersion, the smaller the average nanoparticles size, hence the more frequent Au-rich nanoparticles and the higher the Au/Pd ratio. TEM analyses of our samples corroborate this explanation (Figure S4). The median particle sizes for catalysts prepared with acid were found to be lower than their homologues prepared without acid (Table 7). TEM analysis of the unsupported sol showed a similar trend, with a smaller PSD observed in the presence of acid. When immobilized, the sub-2 nm particles observed in the unsupported sol (in both the presence and absence of acid) are no longer detected in the immobilized sol in the absence of acid but are observed (but less frequently) in the acid system. This suggests that sub-2 nm particles agglomerate during immobilization, resulting in the larger observed PSD for the immobilized catalyst.

Beyond the enhancement of the immobilization fraction of the PAA-stabilized nanoparticles onto the carbon supports during the catalyst preparation, adding acid to the colloidal suspension leads to a better nanoparticle dispersion onto the carbon supports. More active metal sites are available for the reaction and their morphology is more suitable for the H_2_O_2_ synthesis, resulting in enhanced catalytic performances.

The same trend was observed previously by our group by applying an acid pretreatment to carbon-based supports.^14^ AuPd nanoparticles supported on carbons had an enhanced dispersion compared to nontreated supports, resulting in increased activity and selectivity toward the direct synthesis of hydrogen peroxide. However, these catalysts were prepared *via* a wet coimpregnation method, whose chemistry differs largely from the sol immobilization procedure. During the wet impregnation preparation, nanoparticles are formed during the heat treatment of the catalyst precursor *via* the coalescence of dispersed metal salts at the surface of the support. Conversely, during sol immobilization, nanoparticles are formed independently of the support before being immobilized on it. Tiruvalam *et al.* compared the size and the composition of PVA-stabilized AuPd nanoparticles before and after their immobilization on carbon supports. They observed no significant change in nanoparticle composition and explained the small increase in the mean nanoparticle size by the sintering of ultra-small nanoclusters upon immobilization and drying.^16^ Therefore, modifications of nanoparticles should likely occur when the nanoparticles are still dispersed in the preparation solvent, in the colloidal state.

The influence of the acid addition on the catalytic stability has been studied (Table 8). If all catalysts deactivate on reuse, the decrease in activity is less pronounced when catalysts have been prepared with acid. For example, 1%AuPd/KBB-H^+^ loses only 28% of its activity toward the direct synthesis of H_2_O_2_ when reused compared to 90% for 1%AuPd/KBB. This is observed for both H_2_O_2_ production and degradation, regardless of the support. Decreases in H_2_O_2_ production and degradation rates are not correlated, which could support the theory of the different active sites for each reaction.^14^

**Table 8:** Catalytic Testing Results upon Reuse, Metal Leaching and XPS-Derived Au/Pd of PAA-Stabilized 1%AuPd/C Catalyst Prepared with or without Acid and Corresponding Metal Leaching (Au, Pd)^a^

					leaching (%)		
catalyst	use (−)	actual metal loading (wt %)	productivity^b^ (mol_H2O2_ mmol^–1^_Metal_ h^–1^)	degradation^c^ (mol_H2O2_ mmol^–1^_Metal_ h^–1^)	Au	Pd	Au/Pd (−)
1%AuPd/GNP	1	0.48	1.9	1.7	0	0.06	0.2
	2		0.1	0.3			0.2
1%AuPd/GNP-H^+^	1	1.00	2.8	6.4	0	0.04	0.8
	2		0.9	3.1			0.8
1%AuPd/KBB	1	0.21	1.2	3.3	0	0.12	1.1
	2		0.1	0.1			N/A^d^
1%AuPd/KBB-H^+^	1	1.00	1.9	7.3	0	0.10	1.5
	2		1.4	2.8			1.3

a Metal leaching calculations are based on the actual metal loading of the catalyst.

b H_2_O_2_ direct synthesis reaction conditions: catalyst (0.01 g), H_2_O (2.9 g), MeOH (5.6 g), 5%H_2_/CO_2_ (2.9 MPa), 25%O_2_/CO_2_ (1.2 MPa), 0.5 h, 2 °C, 1200 rpm.

c H_2_O_2_ degradation reaction conditions: catalyst (0.01 g), H_2_O_2_ (50 wt % 0.68 g), H_2_O (2.22 g), MeOH (5.6 g), 5% H_2_/CO_2_ (2.9 MPa), 0.5 h, 2 °C, 1200 rpm.

d No Pd detected.

Metal leaching is known to occur for AuPd catalysts on a range of supports.^12,23,66^ Pritchard *et al.* reported the deactivation of both AuPd/C (−26%) and AuPd/TiO_2_ catalysts (−25%) when tested toward the direct synthesis of hydrogen peroxide, with this decrease in activity attributed to the insufficient heat treatment applied (200 °C), likely resulting in metal leaching.^23^ Elemental analyses by ICP-MS reveal no presence of Au in the post-reaction media, in contrast to Pd. The relative amounts of leached metal, with respect to the actual metal loading, are similar for a given support regardless of the acid addition. Interestingly, activity drops are not proportional to the metal leaching, suggesting that deactivation is not only due to the metal loss.

Upon reuse, no significant change in the Au/Pd ratio is observed for GNP-supported catalysts (Table 8), suggesting no change in the composition of the nanoparticles at the catalyst surface. The Au/Pd ratio of 1%AuPd/KBB-H^+^ slightly decreased after the catalytic reaction, which could be explained by the preferential leaching of small Pd clusters to Au (beyond the detection limits of the XRD). Partial oxidation of Pd^0^ to Pd^2+^ occurs during the reaction, which has already been observed for Pd-based catalysts.^67,68^ Since several studies report Pd^2+^ as beneficial for the direct synthesis of hydrogen peroxide,^69−72^ it is unlikely that catalyst deactivation is due to the oxidation of the metal.

We extended our study on the influence of acid on the catalytic activity of AuPd-based catalysts supported on carbon prepared using a series of stabilizers. All catalysts depict similar diffraction patterns regardless of the nature of the stabilizer (Figure S5). As discussed previously, no reflections related to Au, Pd, or PdO were observed but a broad peak appears at 2θ = 39°, suggesting that nanoparticles are randomly alloyed and well dispersed. Catalytic testing results and XPS-derived Au/Pd ratios are summarized in Table 9. All metals are observed to be in the metallic state.

**Table 9:** Catalytic Testing Results and XPS-Derived Au/Pd Molar Ratios of 1%AuPd/C Catalysts Depending on the Stabilizer Used during the Preparation and the Acid Addition

stabilizer	catalyst	productivity^a^ (mol_H2O2_ mmol^–1^_Metal_ h^–1^)	degradation^b^ (mol_H2O2_ mmol^–1^_Metal_ h^–1^)	Au/Pd^c^ (−)
PAA	1%AuPd/GNP	1.9	1.7	0.2
	1%AuPd/GNP-H^+^	2.8	6.4	0.8
	1%AuPd/KBB	1.2	3.3	1.1
	1%AuPd/KBB-H^+^	1.9	7.3	1.5
PVA	1%AuPd/GNP	1.7	3.4	1.0
	1%AuPd/GNP-H^+^	2.1	5.2	1.2
	1%AuPd/KBB	1.2	4.4	1.3
	1%AuPd/KBB-H^+^	2.6	6.8	1.6
SPSS	1%AuPd/GNP	0.7	2.3	1.3
	1%AuPd/GNP-H^+^	1.5	2.4	1.7
	1%AuPd/KBB	d	d	d
	1%AuPd/KBB-H^+^	0.6	3.8	2.2
PDDA	1%AuPd/GNP	7.4	3.6	1.1
	1%AuPd/GNP-H^+^	d	d	d
	1%AuPd/KBB	1.3	4.4	1.3
	1%AuPd/KBB-H^+^	1.4	7.4	1.4
SF	1%AuPd/GNP	2.6	2.9	1.0
	1%AuPd/GNP-H^+^	2.6	2.4	1.0
	1%AuPd/KBB	1.5	7.9	0.6
	1%AuPd/KBB-H^+^	2.1	10.8	1.3

a H_2_O_2_ direct synthesis reaction conditions: catalyst (0.01 g), H_2_O (2.9 g), MeOH (5.6 g), 5%H_2_/CO_2_ (2.9 MPa), 25%O_2_/CO_2_ (1.2 MPa), 0.5 h, 2 °C, 1200 rpm.

b H_2_O_2_ degradation reaction conditions: catalyst (0.01 g), H_2_O_2_ (50 wt % 0.68 g), H_2_O (2.22 g), MeOH (5.6 g), 5% H_2_/CO_2_ (2.9 MPa), 0.5 h, 2 °C, 1200 rpm.

c XPS-derived molar ratios.

d Not applicable as no metal was immobilized.

Catalytic activity depends on the support, the stabilizer, and the acid addition. Changing the nature of the stabilizer influences the catalytic activity of the final material, likely due to structural and/or electronic modifications of the metal nanoparticles and the solid–liquid–gas interface.^18,73−78^ Testing unsupported AuPd nanoparticles (Table S4) highlights the crucial role of both the stabilizer and support in the determination of the catalyst activity through modifications of the metal–support–stabilizer interface.^79^ Regardless of the stabilizer used, catalysts prepared with acid (H_2_SO_4_, 133 ppm) are systematically more active, on a metal molar basis, than their homologues prepared in the absence of acid. The same trend is observed for the Au/Pd molar ratio. The composition of AuPd nanoparticles supported on carbon (Aldrich G60) prepared by sol immobilization has been reported to vary depending on their size. Smaller nanoparticles are Au-rich compared to larger, Pd-rich nanoparticles.^61^ A higher Au/Pd molar ratio, therefore, indicates a higher population of smaller nanoparticles, as demonstrated above for PAA-stabilized AuPd/C catalysts. As a result of their higher metal dispersion, catalysts prepared with acid (H_2_SO_4_, 133 ppm) are more active for H_2_O_2_ synthesis, with lower H_2_O_2_ degradation rates.

Regardless of the nature of the stabilizer, catalysts tend to deactivate upon reuse (Table S5). No significant trend is observed between stabilizers and/or the support. As discussed previously, the low Pd leaching rate is unlikely to be the only reason for the loss of catalytic activity.

## Conclusions

In summary, we have investigated the effect of several parameters (acid addition, nature of the stabilizer, and nature of the support) for the preparation of AuPd catalysts *via* sol immobilization. Metal immobilization fractions are mostly dictated by electronic interactions between the suspended nanoparticles and the support, which are directly governed by the pH of the preparation. Therefore, the ternary system stabilizer-support-pH must be carefully controlled to ensure full metal immobilization. Moreover, adding acid during the preparation influences the size of nanoparticles supported on carbon, increasing the activity of the final catalyst toward the direct synthesis of hydrogen peroxide and its subsequent degradation. We expect this study to help researchers adopt relevant experimental conditions for the preparation of enhanced catalysts designed for many applications requiring supported nanoparticles.

## Supporting Information Available

Extended catalyst testing data, reusability experiments, and characterization data (XRD, DLS, and TEM) (PDF)

## Notes

Information on the data underpinning the results presented here, including how to access them, can be found in the Cardiff University data catalogue at http://doi.org/10.17035/d.2022.0163528087.
