# Supplementary material for: Impact of the Experimental Parameters on Catalytic Activity When Preparing Polymer Protected Bimetallic Nanoparticle Catalysts on Activated Carbon
Source: ACS Catal. 2022 Mar 30;12(8):4440–54. doi: 10.1021/acscatal.1c05904 (PMC9016708; doi:10.1021/acscatal.1c05904)
Supplement: Supplementary file 1 — cs1c05904_si_001.pdf [file cs1c05904_si_001.pdf]

Impact of the Experimental Parameters on Catalytic Activity when Preparing Polymer Protected Bimetallic Nanoparticle Catalysts on Activated Carbon

Electronic Supporting Information

Charlie B. Paris,[a] Alexander G. Howe,[b] Richard James Lewis,[a,c] Daniel Hewes,[a,c] David J. Morgan,[a,d] Qian He,[b] and Jennifer K. Edwards,[a] *

[a] Cardiff Catalysis Institute (CCI), School of Chemistry, Cardiff University, Main Building, Park Place, Cardiff, CF10 3AT, UK.

[b] Department of Materials Science and Engineering, Faculty of Engineering, National University of Singapore, Blk E2, #05-01, 9 Engineering Drive 1, Singapore.

[c] Max Planck centre for Fundamental Heterogeneous Catalysis (FUNCAT), Cardiff Catalysis Institute, School of Chemistry, Cardiff University, Main Building, Park Place, Cardiff, CF10 3AT, United Kingdom.

[d] HarwellXPS - the EPSRC National Facility for Photoelectron Spectroscopy, Research Complex at Harwell (RCaH), Didcot, Oxon. OX11 0FA

* EdwardsJK@cardiff.ac.uk

Table S1 Catalytic testing results for PAA-stabilized AuPd/C catalysts, towards the direct synthesis of H_2O_2 and its subsequent degradation, upon increasing H_2SO_4 (98 wt.%) volume added during the catalyst preparation. The acid volume is as stated.

Support	H_2SO_4 Volume (μL)	H_2SO_4 Concentration (ppm)	Productivity[a] (mol_{H2O2} h^{-1} kg^{-1}_{cat})	Degradation[b] (mol_{H2O2} h^{-1} kg^{-1}_{cat})	Immobilized Fraction (%)	
					Au	Pd
Alfa GNP	0	0	62	55	49	46
	5	43	154	340	100	100
	10	86	151	472	100	100
	15	133	202	460	100	100
	25	215	161	488	100	100
	50	429	217	487	100	100
	100	859	227	471	100	100
	200	1717	225	487	100	100
	500	4293	241	481	100	100
	1000	8587	210	516	100	100
KBB	0	0	17	46	22	20
	5	43	169	174	84	80
	10	86	174	267	97	97
	15	133	141	529	100	100
	25	215	158	596	100	100
	50	429	101	807	100	100
	100	859	106	787	100	100
	200	1717	107	720	100	100
	500	4293	106	633	100	100
	1000	8587	111	624	100	100

[a] **H_2O_2 direct synthesis reaction conditions**: Catalyst (0.01 g), H_2O (2.9 g), MeOH (5.6 g), 5%H_2/CO_2 (2.9), 25% O_2/CO_2 (1.2 MPa), 0.5 h, 2 °C, 1200 rpm. [b] **H_2O_2 degradation reaction conditions**: Catalyst (0.01 g), H_2O_2 (50 wt% 0.68 g), H_2O (2.22 g), MeOH (5.6 g), 5 % H_2/CO_2 (2.9 MPa), 0.5 h, 2 °C, 1200 rpm.

Table S2 Catalytic testing results for PAA-stabilized 1%AuPd/C catalysts, towards the direct synthesis of hydrogen peroxide and its subsequent degradation, upon addition of an equimolar amount (0.27 mmol) of different acids.

Support	Acid	Productivity[a] (mol_{H2O2} h^{-1} kg^{-1}_{cat})	Degradation[b] (mol_{H2O2} h^{-1} kg^{-1}_{cat})	Immobilized Fraction (%)	
				Au	Pd
Alfa GNP	None	62	55	49	46
	H_2SO_4	202	460	100	100
	HNO_3	191	552	100	100
	H_3PO_4	170	474	99	99
	AcOH	142	563	90	88
KBB	None	17	46	22	19
	H_2SO_4	150	529	100	100
	HNO_3	167	481	100	100
	H_3PO_4	128	462	86	78
	AcOH	65	67	46	44

[a] **H_2O_2 direct synthesis reaction conditions**: Catalyst (0.01 g), H_2O (2.9 g), MeOH (5.6 g), 5%H_2/CO_2 (2.9), 25% O_2/CO_2 (1.2 MPa), 0.5 h, 2 °C, 1200 rpm. [b] **H_2O_2 degradation reaction conditions**: Catalyst (0.01 g), H_2O_2 (50 wt% 0.68 g), H_2O (2.22 g), MeOH (5.6 g), 5 % H_2/CO_2 (2.9 MPa), 0.5 h, 2 °C, 1200 rpm.

Table S3 Catalytic testing results for PAA-stabilized mono- and bimetallic AuPd/C catalysts towards the direct synthesis of hydrogen peroxide and its subsequent degradation.

Catalyst	Productivity [a] (mol_{H2O2} $mmol^{-1}_{Metal}$ h^{-1})	Degradation [b] (mol_{H2O2} $mmol^{-1}_{Metal}$ h^{-1})	H_2O_2 Selectivity (%)
1%AuPd/GNP-H^+	202	460	38
1%Au/GNP-H^+	4	22	n/a*
1%Pd/GNP-H^+	23	65	20
1%Au/GNP-H^+ + 1%Pd/GNP-H^+	9	39	n/a*
1%AuPd/KBB-H^+	141	529	25
1%Au/KBB-H^+	3	23	n/a*
1%Pd/KBB-H^+	32	90	11
1%Au/KBB-H^+ + 1%Pd/KBB-H^+	14	59	n/a*

* Not applicable because yields too low. [a] **H_2O_2 direct synthesis reaction conditions**: Catalyst (0.01 g), H_2O (2.9 g), MeOH (5.6 g), 5%H_2/CO_2 (2.9), 25% O_2/CO_2 (1.2 MPa), 0.5 h, 2 °C, 1200 rpm. [b] **H_2O_2 degradation reaction conditions**: Catalyst (0.01 g), H_2O_2 (50 wt% 0.68 g), H_2O (2.22 g), MeOH (5.6 g), 5 % H_2/CO_2 (2.9 MPa), 0.5 h, 2 °C, 1200 rpm.

Table S4 Catalytic testing results for stabilized AuPd colloids towards the direct synthesis of hydrogen peroxide and its subsequent debragadation, with or without acid.

Catalyst	Productivity (a) (mol_{H2O2} kg^{-1}_{cat} h^{-1})	Degradation (b) (mol_{H2O2} kg^{-1}_{cat} h^{-1})
PAA-stab. AuPd colloid	138	710
PAA-stab. AuPd colloid – H+	198	478
PVA-stab. AuPd colloid	138	875
PVA-stab. AuPd colloid – H+	190	669
SPSS-stab. AuPd colloid	117	302
SPSS-stab. AuPd colloid – H+	141	280
PDDA-stab. AuPd colloid	21	484
PDDA-stab. AuPd colloid – H+	30	267
Stabilizer-free AuPd colloid	118	856
Stabilizer-free AuPd colloid – H+	182	806

[a] **H_2O_2 direct synthesis reaction conditions**: Catalyst (0.01 g), H_2O (2.9 g), MeOH (5.6 g), 5%H_2/CO_2 (2.9), 25% O_2/CO_2 (1.2 MPa), 0.5 h, 2 °C, 1200 rpm. [b] **H_2O_2 degradation reaction conditions**: Catalyst (0.01 g), H_2O_2 (50 wt% 0.68 g), H_2O (2.22 g), MeOH (5.6 g), 5 % H_2/CO_2 (2.9 MPa), 0.5 h, 2 °C, 1200 rpm.

Table S5 Reusability study for 1%AuPd/C catalysts and metal leaching. Metal leaching calculations are based on the actual metal loading of the catalyst.

Support	Stabilizer	Acid addition	Actual Metal Loading (wt.%)	Use (-)	Productivity [(a)] ($mol_{H2O2}\ kg^{-1}_{cat}\ h^{-1}$)	Degradation [(b)] ($mol_{H2O2}\ kg^{-1}_{cat}\ h^{-1}$)	Leaching (%) Au	Pd
GNP	PAA	Yes	0.48	1	62	55	BDL	0.06
			-	2	0	10	-	-
		No	1.00	1	202	460	BDL	0.08
			-	2	68	226	-	-
	PVA	Yes	0.41	1	90	316	BDL	0.07
			-	2	68	179	-	-
		No	0.99	1	151	369	BDL	0.05
			-	2	100	150	-	-
	SPSS	Yes	0.55	1	29	91	/ *	/ *
			-	2	8	0	-	-
		No	0.96	1	103	165	/ *	/ *
			-	2	30	80	-	-
	PDDA	Yes	0.06	1	21	67	/ *	/ *
			-	2	3	11	-	-
		No	0.00	1	0	0	N/A	N/A
			-	2	0	0	-	-
	SF	Yes	1.00	1	159	209	BDL	BDL
			-	2	30	176	-	-
		No	1.00	1	186	176	BDL	0.02
			-	2	31	53	-	-
KBB	PAA	Yes	0.21	1	17	46	BDL	0.12
			-	2	3	0	-	-
		No	1.00	1	141	529	BDL	0.10
			-	2	101	205	-	-
	PVA	Yes	1.00	1	89	202	BDL	0.08
			-	2	31	102	-	-
		No	0.98	1	186	529	BDL	0.16
			-	2	82	205	-	-
	SPSS	Yes	0.00	1	0	0	N/A	N/A
			-	2	0	0	-	-
		No	0.36	1	16	98	/ *	/ *

		-	2	5	22	-	-
PDDA	Yes	1.00	1	87	259	/ *	/ *
		-	2	64	191	-	-
	No	0.96	1	97	512	/ *	/ *
		-	2	51	159	-	-
SF	Yes	1.00	1	112	570	BDL	BDL
		-	2	97	262	-	-
	No	1.00	1	155	782	BDL	0.09
		-	2	119	127	-	-

BDL = Below detection limit. * Analysis not performed. **(a) H_2O_2 direct synthesis reaction conditions**: Catalyst (0.01 g), H_2O (2.9 g), MeOH (5.6 g), 5%H_2/CO_2 (2.9 MPa), 25%O_2/CO_2 (1.2 MPa), 0.5 h, 2 °C, 1200 rpm. **(b) H_2O_2 degradation reaction conditions**: Catalyst (0.01 g), H_2O_2 (50 wt% 0.68 g), H_2O (2.22 g), MeOH (5.6 g), 5 % H_2/CO_2 (2.9 MPa), 0.5 h, 2 °C, 1200 rpm.

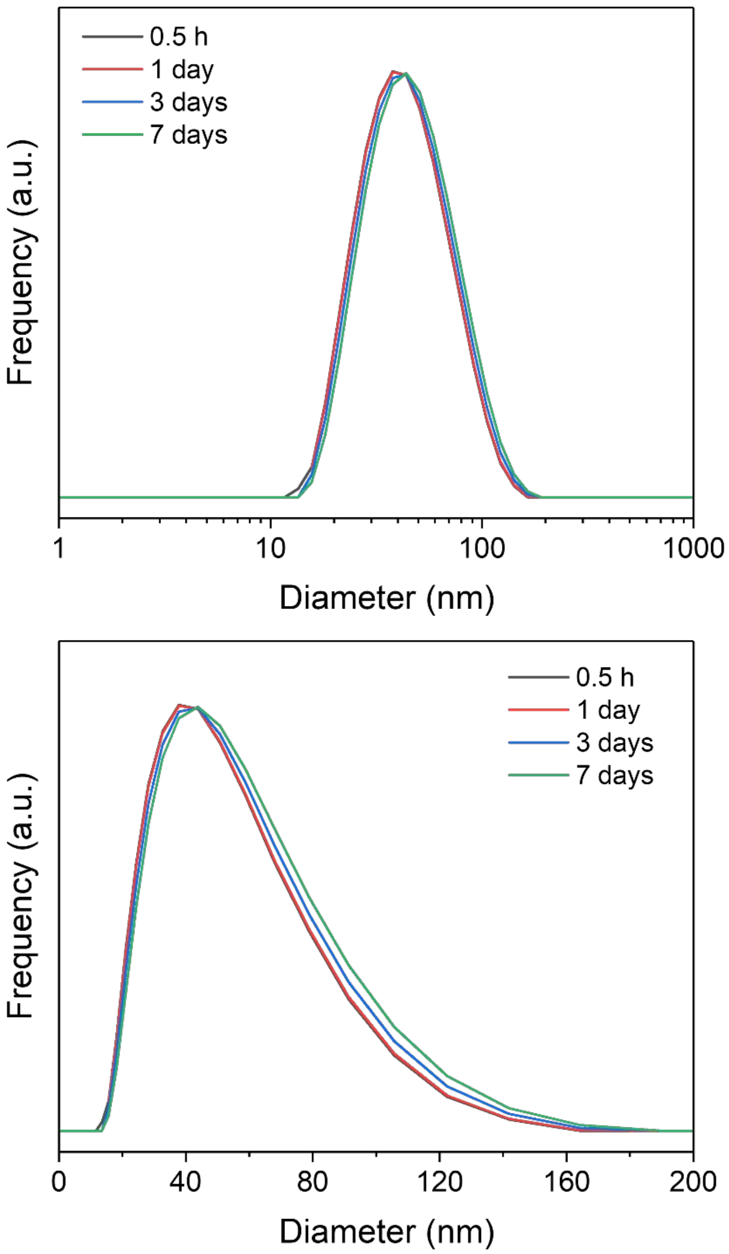

Figure S1 Particle size distribution by frequency of PAA-stabilized AuPd nanoparticles, prepared without acid, over time.

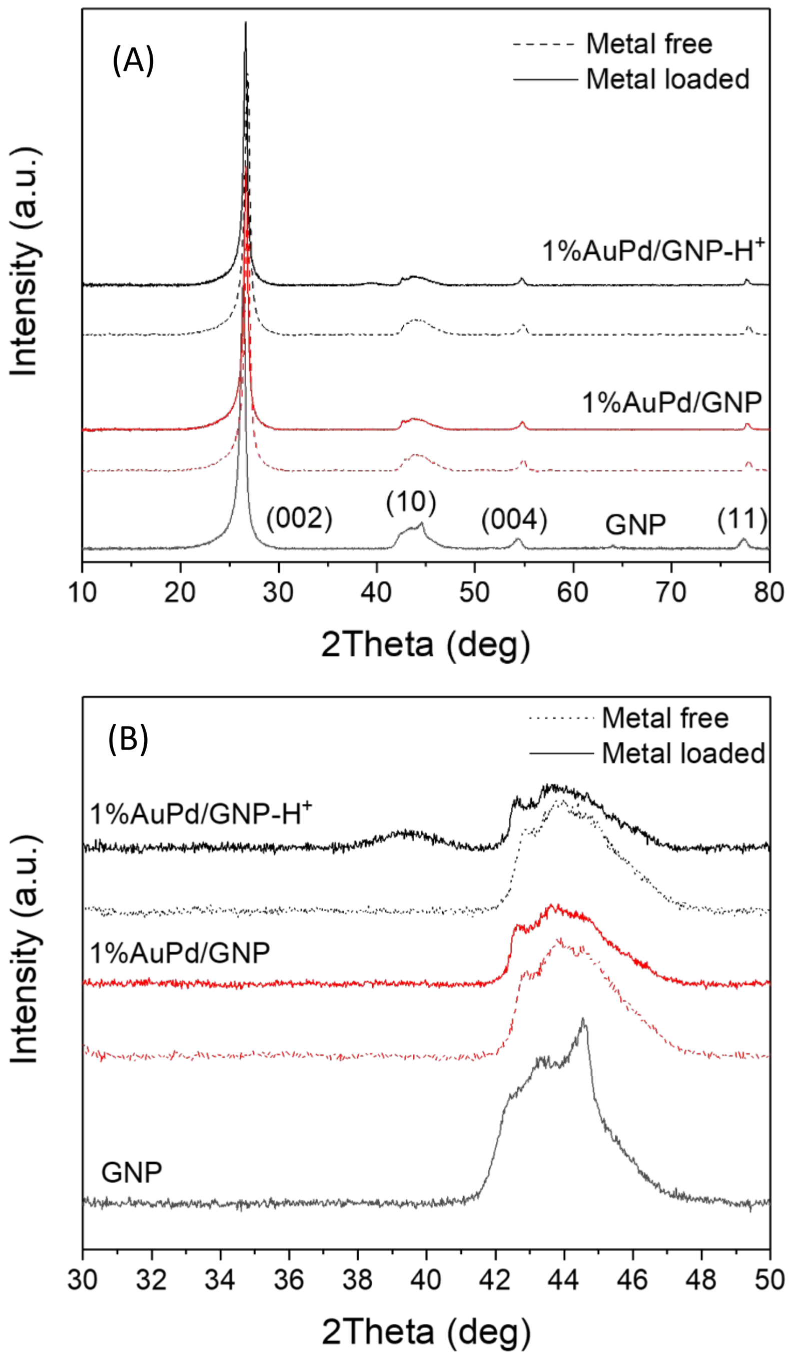
(A)
Metal free
Metal loaded
Intensity (a.u.)
1%AuPd/GNP-H+
1%AuPd/GNP
(002)
(10)
(004)
GNP
(11)
10 20 30 40 50 60 70 80
2Theta (deg)
(B)
Metal free
Metal loaded
1%AuPd/GNP-H+
1%AuPd/GNP
GNP
Intensity (a.u.)
30 32 34 36 38 40 42 44 46 48 50
2Theta (deg)

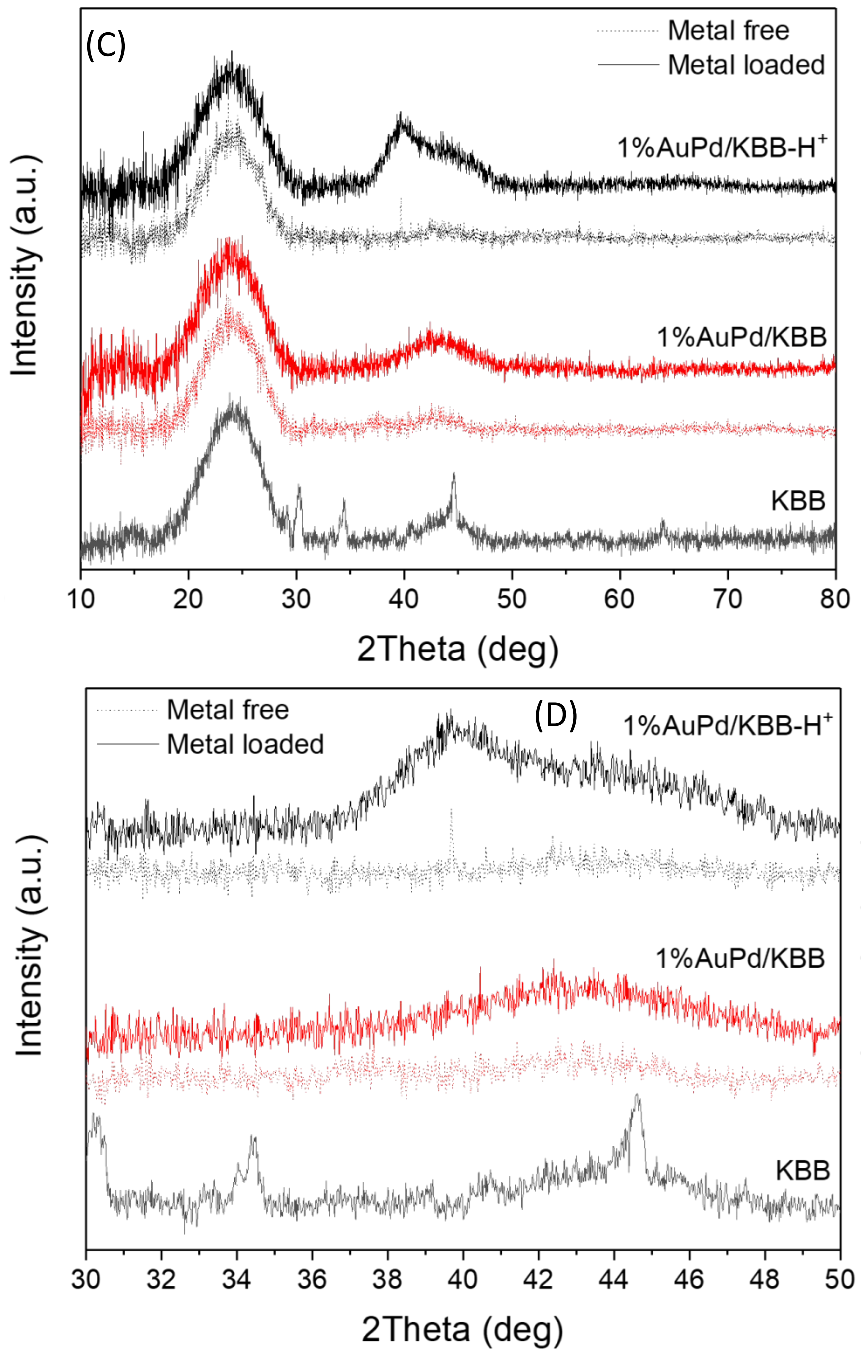

Figure S2 Diffractograms of pristine supports (grey), metal-free catalysts (dotted line) and 1%AuPd/C catalysts (full line), with (black) or without (red) acid. (A) and (B): 1%AuPd/GNP ; (C) and (D): 1%AuPd/KBB

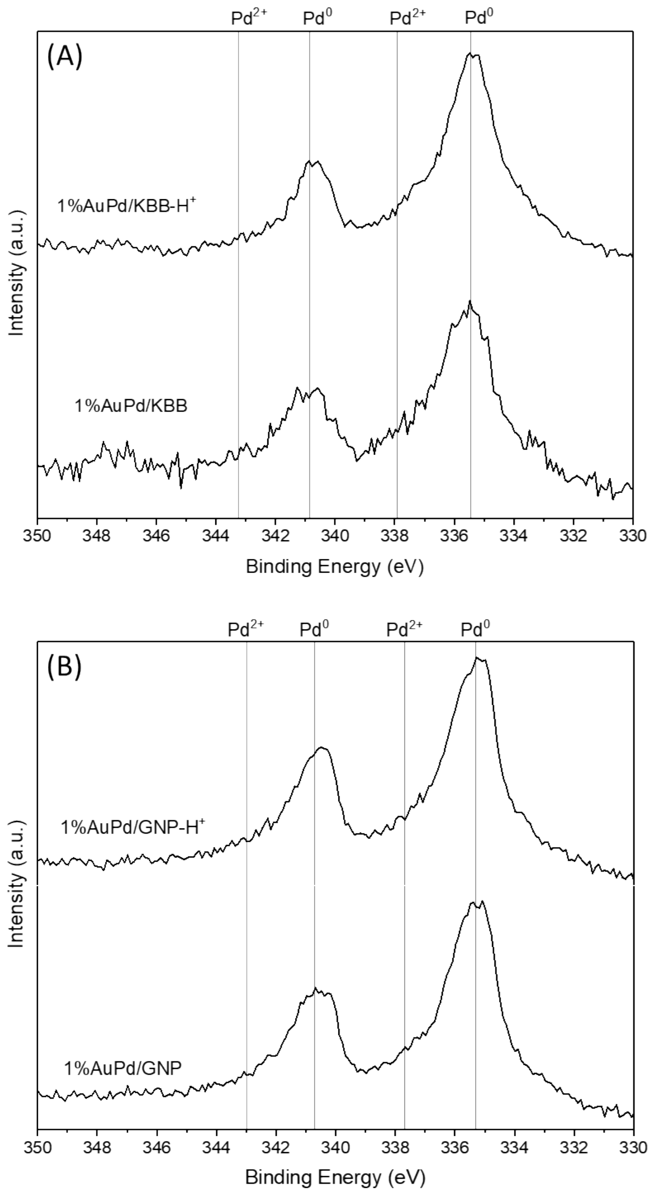

Figure S3 Pd(3d) spectra of (A) 1%AuPd/GNP(-H^+) and (B) 1%AuPd/KBB(-H^+).

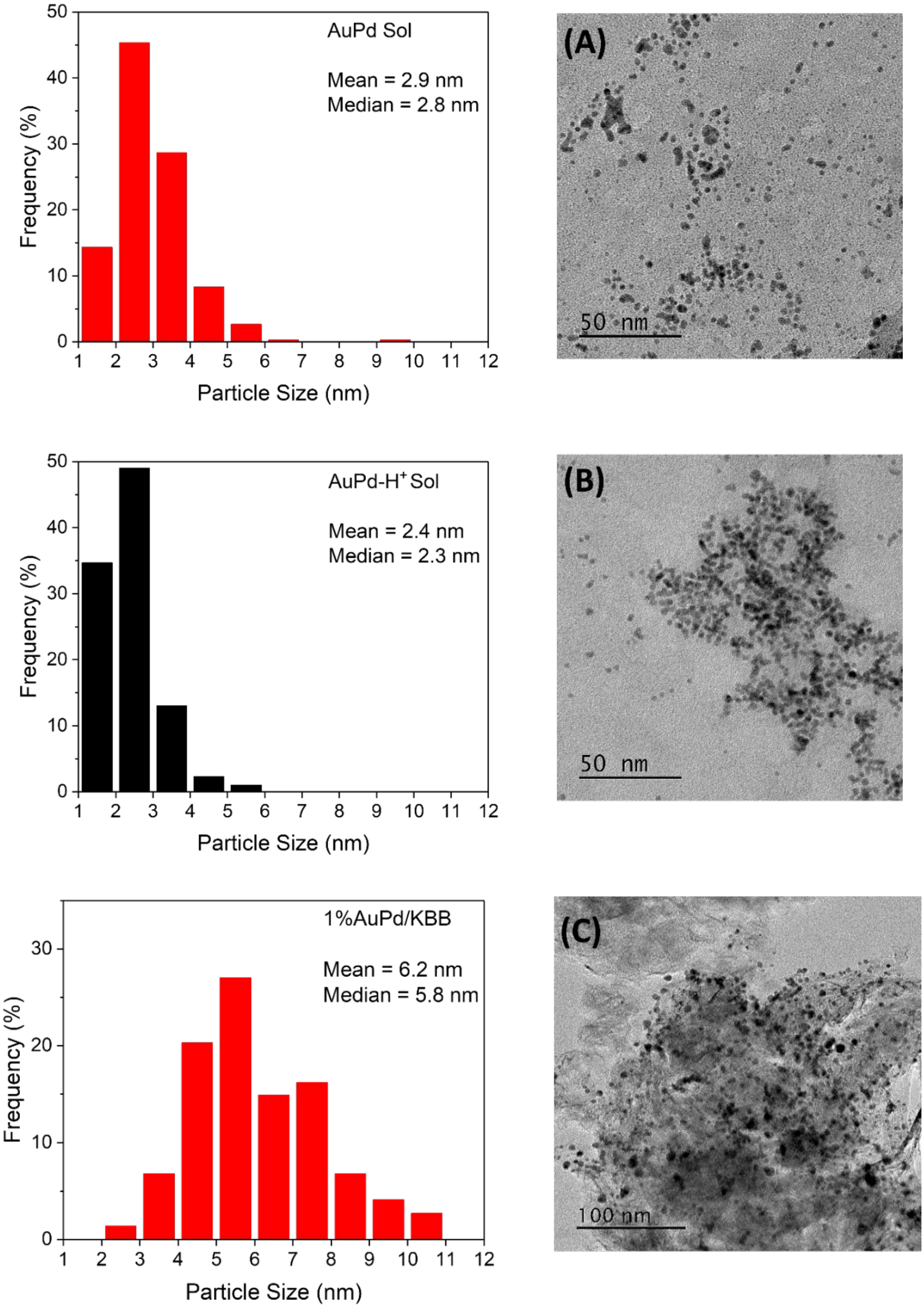
AuPd Sol
Mean = 2.9 nm
Median = 2.8 nm
Frequency (%)
Particle Size (nm)
(A)
50 nm
AuPd-H+ Sol
Mean = 2.4 nm
Median = 2.3 nm
Frequency (%)
Particle Size (nm)
(B)
50 nm
1%AuPd/KBB
Mean = 6.2 nm
Median = 5.8 nm
Frequency (%)
Particle Size (nm)
(C)
100 nm

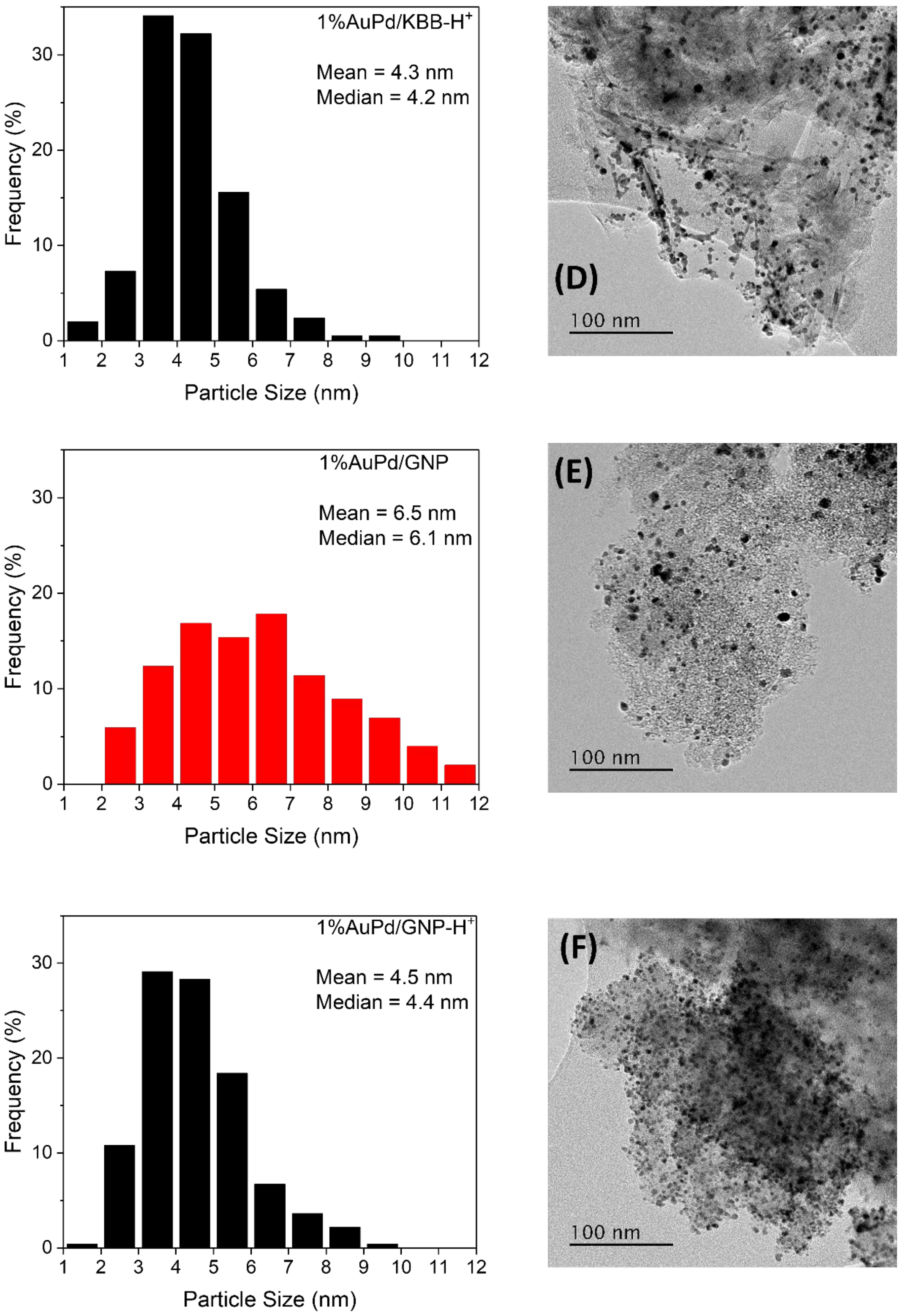

Figure S4 Nanoparticle size distribution and TEM images of (A) unsupported AuPd sol, (B) unsupported AuPd acidified sol, (C) 1%AuPd/KBB, (D) 1%AuPd/KBB-H^+, (E) 1%AuPd/GNP, and (F) 1%AuPd/GNP-H^+.

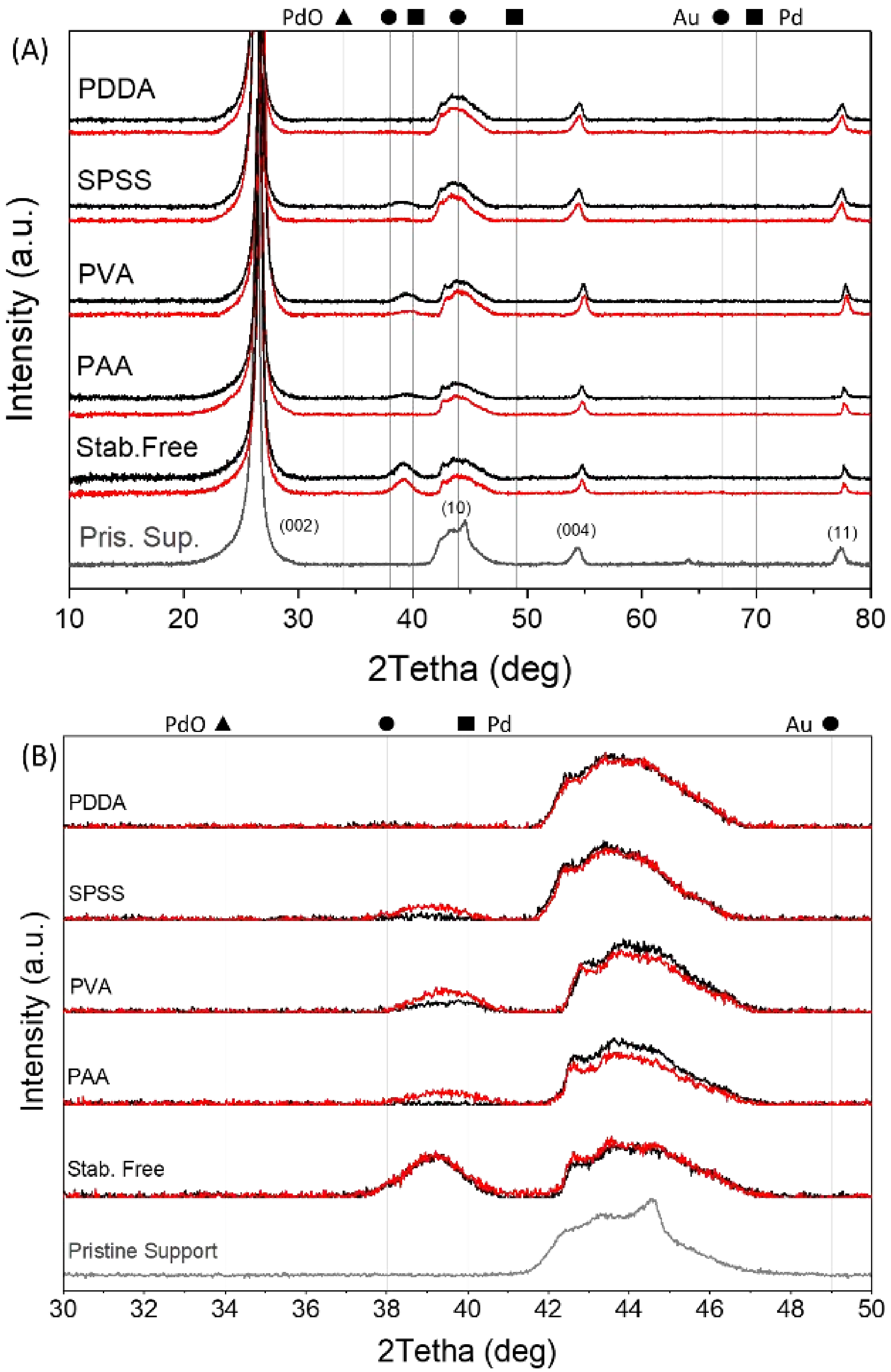

PdO
Au
Pd
(A)
PDDA
SPSS
PVA
PAA
Stab.Free
Pris. Sup.
(002)
(10)
(004)
(11)
Intensity (a.u.)
10
20
30
40
50
60
70
80
2Tetha (deg)
(B)
PDDA
SPSS
PVA
PAA
Stab. Free
Pristine Support
Intensity (a.u.)
30
32
34
36
38
40
42
44
46
48
50
2Tetha (deg)

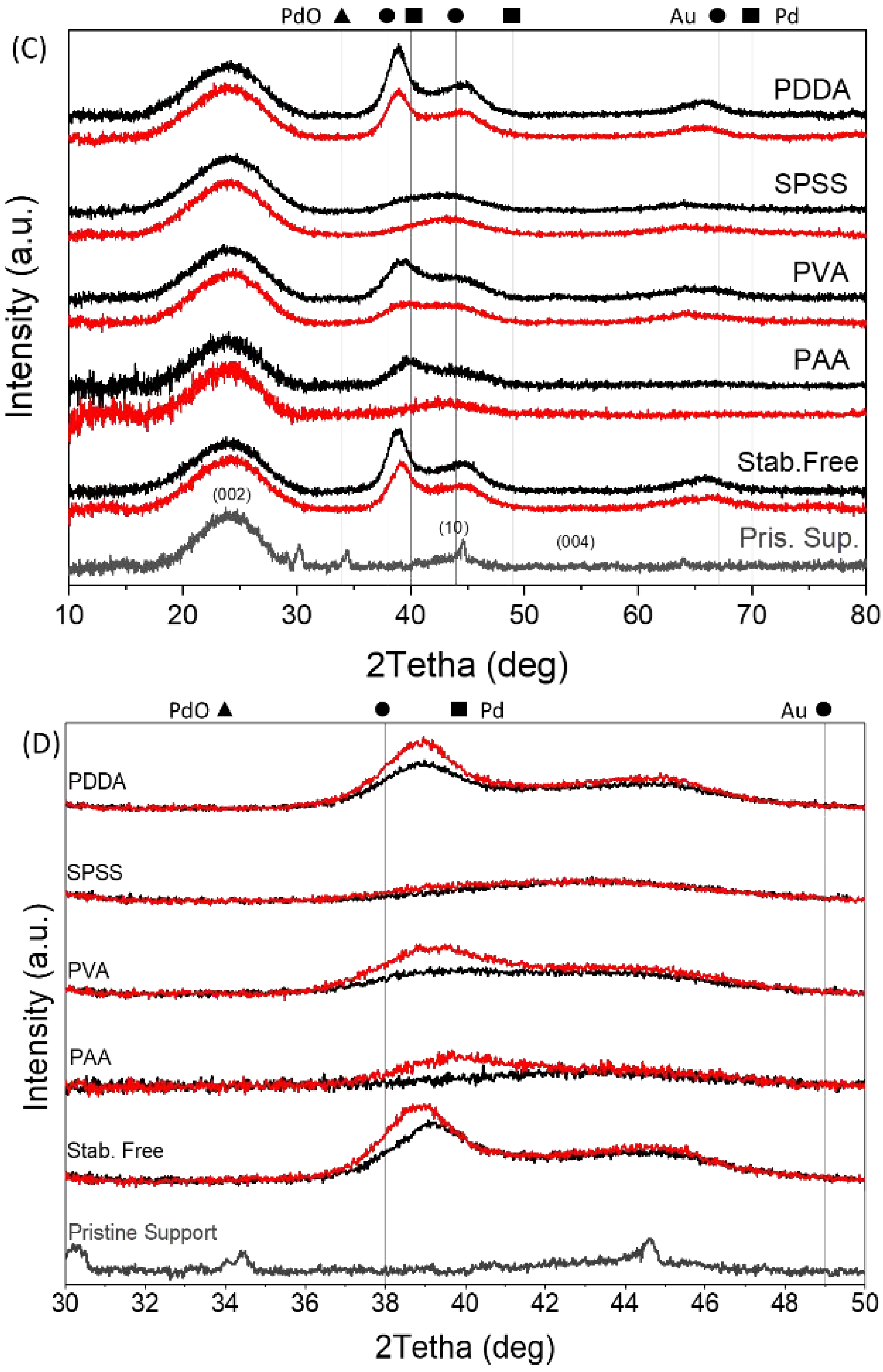

Figure S5 Diffractograms of pristine supports (grey) and 1%AuPd/C catalysts prepared using a series of stabilizer, with (black) or without (red) acid. (A) and (B): 1%AuPd/GNP ; (C) and (D): 1%AuPd/KBB
